# Allelic Variants of HLA-C Upstream Region, *PSORS1C3*, *MICA*, *TNFA* and Genes Involved in Epidermal Homeostasis and Barrier Function Influence the Clinical Response to Anti-IL-12/IL-23 Treatment of Patients with Psoriasis

**DOI:** 10.3390/vaccines10111977

**Published:** 2022-11-21

**Authors:** Martina Morelli, Marco Galluzzo, Claudia Scarponi, Stefania Madonna, Giovanni Luca Scaglione, Giampiero Girolomoni, Marina Talamonti, Luca Bianchi, Cristina Albanesi

**Affiliations:** 1 Laboratory of Experimental Immunology, IDI-IRCCS, 00167 Rome, Italy; 2 Dermatology Unit, Fondazione Policlinico “Tor Vergata” and Department of Systems Medicine, “Tor Vergata” University of Rome, 00133 Rome, Italy; 3 Section of Dermatology and Venereology, Department of Medicine, University of Verona, 37126 Verona, Italy

**Keywords:** psoriasis, SNP, HLA-C, anti-IL-12/23, ustekinumab, pharmacogenomics

## Abstract

Several biologic therapies have been developed to treat moderate-to-severe psoriasis, with patients exhibiting different clinical benefits, possibly due to the heterogeneity of pathogenic processes underlying their conditions. Ustekinumab targets the IL-12/IL-23-p40 subunit and inhibits type-1 and type-17 T-cell responses. Although ustekinumab is effective as both short- and long-term treatment, therapeutic response varies considerably among patients. Ustekinumab biosimilars will be commercialized in the very next future, likely broadening the use of this drug in the treatment of psoriasis patients. Our pharmacogenomic study evaluated the influence of 417 single-nucleotide polymorphisms (SNPs) in psoriasis-risk alleles on the clinical response to ustekinumab in a cohort of 152 patients affected by moderate-to-severe plaque-type psoriasis. Differences in SNP pattern characterizing HLA-Cw6^+^ or HLA-Cw6^−^ patients, showing high or low responses to ustekinumab, were also analysed. We identified twelve SNPs in HLA-C upstream region (rs12189871, rs4406273, rs9348862 and rs9368670), *PSORS1C3* (rs1265181), *MICA* (rs2523497), LCE3A-B intergenic region (rs12030223, rs6701730), *CDSN* (rs1042127, rs4713436), *CCHCR1* (rs2073719) and in *TNFA* (rs1800610) genes associated with excellent response to ustekinumab. We also found that HLA-Cw6^+^ and HLA-Cw6^−^ patients carried out distinct patterns of SNPs associated with different clinical responses. The assessment of HLA-C alleles, together with other genetic variants, could be helpful for defining patients who better benefit from anti-IL-12/IL-23 therapy.

## 1. Introduction

Psoriasis is a lifelong and clinically heterogeneous skin disease that presents in multiple forms associated with several comorbid conditions [1]. Different pathogenic processes operate in psoriatic skin lesions depending on the clinical phase of the disease. While innate immunity responses predominate in the acute phase, acquired immune responses prevail in the chronic phase of psoriasis, with massive recruitment into skin lesions of myeloid dendritic cells and (auto)reactive T lymphocytes, mostly T helper (Th)17 and type-1 IFN-γ-producing T cells [2]. Acute responses preferentially develop in the early psoriatic lesions, as well as in certain clinical subtypes, including exanthematic and pustular psoriasis. In contrast, chronic responses characterize stable plaques, even though inhomogeneous or evolving plaques can show both acute and chronic inflammatory areas [3].

The heterogeneity of psoriasis is also dependent on the complex interplay of genetic and environmental factors [4,5], and molecular genetics studies have identified more than 65 independent genomic risk loci that confer risk of the disease [6,7,8]. These susceptibility loci can span many genes involved in specific adaptive and innate immune pathways, such as antigen presentation, Th17 cell activation, innate antiviral immunity/type I IFN signaling and skin barrier function [9,10,11,12,13]. Among risk loci, the psoriasis susceptibility 1 (*PSORS1*) locus accounts for 30–50% of disease heritability and spans 179 Kb region on chromosome 6p21.3, within the major histocompatibility complex (MHC) class I region [4,5]. *PSORS1* locus contains at least fifteen genes, including the highly polymorphic human leukocyte antigen (HLA)-C gene encoding a type of heavy chain of HLA class I. The HLA-Cw6 allelic variant of *HLA-C* is highly represented in the psoriatic population (>60%) and is itself considered as the causal susceptibility allele for psoriasis, even though over one-hundred single-nucleotide polymorphisms (SNPs) of HLA-C genic and intergenic region have been described in patients [5]. Importantly, evidence has emerged for the presence of susceptibility alleles of other MHC class I genes and regulatory regions, potentially influencing HLA expression in the psoriatic population. They include polymorphic regions in proximity to MHC class I polypeptide-related gene, which encodes a MHC class I-related protein with potential immunological functions on IL-17A-producing and NKG2D-bearing NK and CD8^+^ T cells [14].

HLA-C risk alleles have been implicated in psoriasis development and progression, since they preferentially present (auto)antigens, such as the LL37 cathelecidin and the disintegrin and metalloprotease domain containing thrombospondin type 1 motif-like 5 (ADAMTSL5) protein, to CD8^+^ T cells with a cytokine and skin-homing receptor profile typical of psoriatic skin (IL-17^high^, IFN-γ^high^, CLA^+^, CCR6^+^ and CCR10^+^) [15,16]. The relevance of HLA-Cw6 in psoriasis pathogenesis has also been confirmed by pharmacogenomic studies showing that its presence in patients associates with better response to the anti-IL-12/IL-23p40 ustekinumab [17,18] or anti-IL-17A secukinumab drugs [19].

Several biologic therapies have been developed to treat moderate-to-severe psoriasis, with patients exhibiting different degree of clinical responses, possibly due to the heterogeneity of pathogenic processes underlying their conditions. Among biologics, ustekinumab targets the p40 subunit of IL-12 and IL-23, two dendritic cell (DC)-derived cytokines driving type-1 and type-17 T cell responses. Although ustekinumab is safe and effective as both short- and long-term therapy, only the 46–63% of patients undergone ustekinumab treatment can achieve a substantial clinical improvement [20,21]. Therefore, the identification of potential predictors for optimal response to ustekinumab could be useful for increasing the success rate with this therapy in clinical practice. Indeed, HLA-Cw6 positivity, female gender, and body mass index (BMI) <30 kg/m^2^ have been identified as predictor variables for improved response to ustekinumab [21].

Here, we report a pharmacogenomic study aimed at evaluating the simultaneous presence of a panel of genetic polymorphisms related to psoriasis-risk loci, associated with clinical response to ustekinumab, in a cohort of 152 patients affected by moderate-to-severe plaque psoriasis. A number of SNPs potentially predicting the response to ustekinumab was identified.

## 2. Materials and Methods

### 2.1. Patients and Ethics Statement

Adult subjects affected by moderate-to-severe plaque-type psoriasis were recruited between September 2015 and June 2018 (*n* = 152) at the Dermatology Unit of Tor Vergata University of Rome. All patients were Caucasians, aged >18 years with, and characterized by: Psoriasis Area Severity Index (PASI) score >10, body surface area (BSA) >10%, dermatology life quality index (DLQI) >10. Patients with a baseline PASI <10, who presented involvement of sensitive areas were also included. The enrolled patients required biologic treatment with ustekinumab, as failed to respond, had contraindications for, or did not tolerate at least one conventional treatment. 

Ustekinumab biologics was administered following AIFA (Agenzia Italiana del Farmaco) criteria in a standard dosing regimen (55 mg for patients ≤100 kg and 90 mg in patients >100 kg at weeks 0, 4 and every 12 weeks thereafter), used in monotherapy, and not combined with conventional systemics or topical therapies. 

For each patient, personal data, as well as anthropometric, socio-demographic and clinical data were collected and annotated in a database *ad hoc* created for the study. The disease severity and response to treatment were evaluated using the PASI score at baseline and at follow-up visits on weeks 4, 12, 28, 40, 52, 64, 76, 88 and 100. Clinical efficacy was defined as the 75%, 90% and 100% improvement of PASI score compared with baseline and termed PASI75, PASI90 and PASI100, respectively. 1-mL blood samples were collected from each psoriatic patient to isolate DNA. 

The study was conducted in accordance with the guidelines of the Declaration of Helsinki and approved by the Tor Vergata University Ethics Committee (approval no. 20745, v. 25 Mar 2005). Thus, clinical data, as well as blood were collected from patients after written informed consent.

### 2.2. SNP Analysis 

DNA was extracted from blood using the QIAcube system with QIAmp DNA kit (Qiagen, Hilden, Germany), and 10 ng were used for sequencing by NGS technology. The customized designed SNP panel was composed of *n* =139 SNPs (Supplementary Table S1), present in *n* =89 amplicons (size range 125–375 bp). This panel permitted to identify 417 genetic variants in total (Supplementary Table S1). The analysed SNPs were selected based on an extensive review of articles on the association between psoriasis and SNPs or response to biologics [7,9,12,18,22,23,24].

NGS was performed using the Ion GeneStudio™ S5 Plus platform (Thermo Fisher Scientific, Massachusetts, USA). Libraries were amplified by the Ion AmpliSeq™ Library kit Plus (Thermo Fisher Scientific) and quantified using the Qubit 4 Fluorometer and 2100 Bioanalyzer with dsDNA HS assay and High Sensitivity DNA kit (Thermo Fisher Scientific), respectively. Sequencing data were processed with the Ion Torrent Suite software v.5.10. 

Positive calls were selected with a read depth >30X and allelic frequency higher than 0.3 (range 0–1.0). Reads were aligned to human genome sequence (build GRCh37/human genome 19). Variants were collected using Variant Caller plugin and systematically evaluated, filtered, and annotated using tailored R scripts. Variants’ annotations were finally verified using ANNOVAR.

### 2.3. Statistical Analysis 

The primary aim of this study was to identify the SNP(s) or clusters of co-segregating SNPs associated with an optimal response to ustekinumab in a patient population of *n* =152 subjects. Sample size has been calculated based on the real-life observations on patients undergone ustekinumab treatment and identifying the achievement of PASI90 at week 52 as primary endpoint [20]. In addition, sample size has been estimated by assuming that relevant SNPs have a prevalence >8% in the psoriatic population and choosing the levels alpha = 0.05 two-sided and beta =0.20 (i. e. power of the study = 80%).

Drug response data were analyzed by an intent-to-treat last observation carried forward method. SNPs that showed an identical pattern in patients have been merged to reduce the number of genetic variables that needed to be managed.

Univariate logistic regression analysis for two parameters (bivariate analysis) was assessed for all SNPs and achievement of PASI75, 90, and 100 at weeks 4, 12, 28, 40, 52, 64, 76, 88 and 100, and presented as odds ratio (OR) and 95% confidence intervals (CI).

Stepwise multivariate logistic regression models were also performed to evaluate the association between SNPs significantly correlating with response to ustekinumab at univariate logistic regression analysis and demographic, anthropometric and clinical characteristics variables (e.g., sex, age, age at onset of disease, disease duration, PASI at baseline, number of comorbidities, and number of previous biologic treatments, achievement of PASI75, 90, and 100 at each time point of observation) to draw a predictive model of PASI response.

The association between drug response and all the variables collected was estimated using the STATA 14.2 software (StataCorp, College Station, TX, USA). Deviation from null hypothesis was considered significant at *p*-value <0.05. 

For drug survival analyses, a time-to-event analysis have been performed. The Kaplan-Meier method has been used to estimate time-to-loss of response, and the log-rank test was applied to compare time estimates between groups. 

Canonical analysis of principal coordinates (CAP), based on the Bray-Curtis similarity matrix [25], was performed to assess multivariate differences in the genetic patterns of psoriatic population among the HLA-Cw6^+^ or HLA-Cw6^−^ patients showing optimal or low clinical response to ustekinumab or BMI ≥30 and ≤30. Genetic patterns were also assessed in patients grouped based on their clinical response rate to ustekinumab. Three groups were established: responders up to 2-year treatment, non-responders or only partially responders for primary failure (not achieving PASI75 after 16 weeks of treatment) and non-responders for secondary failure (loosing PASI75 after 16 weeks of treatment). The canonical correlations were tested using 4999 random permutations of the genetic data, expressed as presence of the SNPs. The analysed data matrix included 94 SNPs, since SNPs showing identical patterns in psoriatic patients were deleted. Moreover, distinctness of the four patient groups (HLA-Cw6^+^ high-responders, HLA-Cw6^+^ low-responders, HLA-Cw6^−^ high-responders, HLA-Cw6^−^ low-responders; HLA-Cw6^+^ BMI > 30, HLA-Cw6^+^ BMI < 30, HLA-Cw6^−^ BMI > 30, HLA-Cw6^−^ BMI < 30) or of the three patient groups showing different responses to the drug (2-year responders, non-responders for primary failure or responders losing responsiveness for secondary failure) was assessed using leave-one-out allocation success [26]. The product-moment correlations of the selected 94 SNPs with the two canonical discriminant axes (ρ1 and ρ2) were calculated, and only the most relevant correlations (i.e., √ρ1^2^ + ρ2^2^ > 0.35) were considered as valuable and included in the plot. The multivariate analysis was carried out using PRIMER 6© v.6.1.5 and PERMANOVA +© v.1.0.1 software (PRIMER-E, Plymouth, UK).

## 3. Results

### 3.1. Patients Responding to Ustekinumab Therapy Show Specific SNP Patterns Coherently with HLA-Cw6 Status and BMI Values < 30

Aimed at identifying 139 SNPs in the DNA of psoriatic patients, we first analysed a customized SNP panel by using NGS technology. Sequencing of amplicons permitted the identification of additional 278 SNPs located nearby the primary SNPs. Thus, we obtained information on 417 allelic variants of psoriasis-risk genes involved in immune responses processes, such as antigen presentation, innate immunity, cytokine-dependent pathways and T-cell signalling, and skin barrier function (Supplementary Table S1). The SNP patterns were analysed in a patient cohort of psoriatic subjects (*n* = 152) whose demographic, anthropometric and disease characteristics are summarized in Table 1. At baseline, mean PASI was 19.1 ± 10.8, disease duration was 21.1 ± 13.4 years, and age at disease onset was 27.1 ± 13.7 years. Mean weight and BMI were 81.2 ± 20.9 and 29.1 ± 17.8, respectively. Most of the patients (55.2%) were naive to biological therapy. Hypertension was the most frequent comorbidity, together with obesity and hyperlipidemia. PASI follow-up scores were available for 123 (79.9%) patients at the central observational point (52 weeks) and for 94 (62.9%) at the last follow-up visit (100 weeks). 

**Figure 1:**
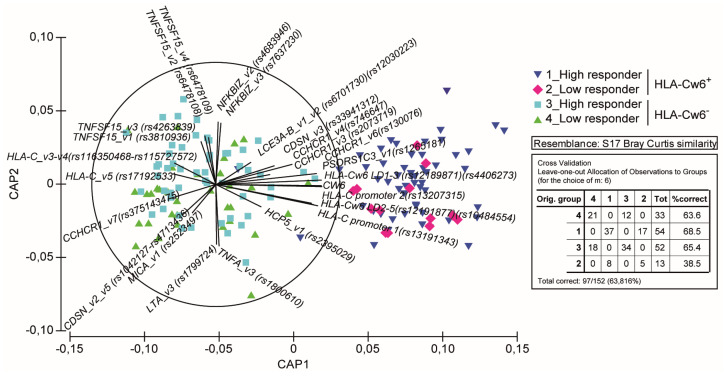
HLA-Cw6 status identifies specific SNP patterns in psoriatic patients associated with clinical response to ustekinumab. Clustering of patients based on presence of SNPs and response to ustekinumab was performed by CAP on a cohort of psoriatic patients (*n* = 152), classified as high responders to ustekinumab (PASI90 or PASI100 achievement up to 2-year treatment) or low responders (non-responders or only partially responders for primary failure or responders losing responsiveness over time for secondary failure). CAP ordination plot shows a significant clustering of psoriatic patients belonging to HLA-Cw6^+^ and HLA-Cw6^−^ groups along *x*-axis, and, in particular, to four established subgroups: 1-HLA-Cw6^+^ high-responders (blue downward triangles), 2-HLA-Cw6^+^ low-responders (fuchsia diamonds), 3- HLA-Cw6^−^ high-responders (light blue squares), and 4- HLA-Cw6^−^ low-responders (green triangles) to ustekinumab. The length of each vector line corresponds to the strength of the correlation and direction for each. Distinctness of the four patient groups was assessed using leave-one-out allocation success. 97 out of 152 (63.82%) patients were correctly allocated. Only the most relevant correlations were considered as valuable and included in the plot.

SNPs and genetic variants were annotated in the database for each patient as wild-type alleles, homo- or heterozygotic variants. Subjects carrying SNPs in homozygosis or heterozygosis were indistinctly considered as SNP-positive subjects. Psoriatic population was then clustered based on HLA-Cw6 presence (*n* = 67) or absence (*n* = 85) and on the clinical response to ustekinumab. Thus, high-responder patients showed an optimal response to ustekinumab up to two years of treatment (*n* = 106), whereas patients who failed to respond or only partially responded to the biologic were considered as low responders (*n* = 46). In order to identify the SNP patterns characterizing patients in relation to HLA-Cw6 allele status and/or high or low clinical responses to ustekinumab, we performed CAP. This analysis showed a significant clustering of patients belonging to HLA-Cw6^+^ and HLA-Cw6^−^ groups, and, specifically, in four established subgroups: HLA-Cw6^+^ high-responders (*n* = 54), HLA-Cw6^+^ low responders (*n* = 13), HLA-Cw6^−^ high responders (*n* = 52), and HLA-Cw6^−^ low-responders (*n* = 33) (Figure 1). HLA-Cw6^+^ high-responders were properly distributed in the higher right quadrant of plot area (37/54), whereas the HLA-Cw6^+^ low-responder cluster was mostly found in the lower right quadrant (8/13). However, few patients of the HLA-Cw6^+^ high- (17/54) and low-responder (5/13) groups did not appropriately allocate in their respective quadrants (Figure 1), likely due to their similar genetic SNP profiles.

Similarly, among HLA-Cw6^−^ patients, 18 out of 52 high-responders and 12 out of 33 low-responders were not found in their clusters. It is noteworthy that the percentage of low-responders of the HLA-Cw6^+^ group was substantially lower than that of HLA-Cw6^−^group (19.1% vs. 38.9%, respectively). As shown in Figure 1, HLA-Cw6^+^ and HLA-Cw6^−^ groups totally segregated along the *x*-axis and showed a distinct pattern of SNP presence. In particular, SNPs of LCE intergenic region (rs12030223, rs6701730), *CDSN* (rs33941312), *CCHCR1* (rs746647 rs2073719 and rs130076), *PSORS1C3* (rs1265181), HLA-C intergenic region (rs12189871, rs4406273, rs12191877, rs10484554), HLA-C promoter region (rs13207315, rs13191343) and *HCP5* (rs2395029) were mostly carried out by HLA-Cw6^+^ patients (Figure 1). On the other hand, the presence of SNPs in *TNFSF15* (rs3810936, rs6478108, rs4263839, rs6478109), *CCHCR1* (rs375143475), *CDSN* (rs1042127, rs4713436), *MICA* (rs2523497) and in HLA-C intergenic region (rs116350468, rs115727572, rs17192533) characterized HLA-Cw6^−^ patients. Interestingly, the pattern of SNPs in HLA-C genic and intergenic regions characterizing HLA-Cw6^+^ and HLA-Cw6^−^ patients were different and mutually exclusive (Figure 1). Of note, rs12030223 and rs6701730 (LCE_v1_v2 intergenic region), rs33941312 (CDSN_v3), rs746647, rs2073719 and rs130076 (CCHCR1_v3, _v6 and _v4), rs1265181 (PSORS1C3_v1), rs12189871 and rs4406273 (HLA-Cw6 LD1 and HLA-Cw6 LD3 intergenic region) characterized HLA-Cw6^+^ patients optimally responding to ustekinumab, whereas variants of *TNFSF15* (rs6478109, rs6478108, rs4263839 and rs3810936), *HLA-C* (rs115350468, rs115727572 and rs17192533) and *CCHCR1* (rs375143475) characterized the HLA-Cw6^−^ high-responder population (Figure 1). 

The presence of CDSN_v2-v5 (rs1042127 and rs4713436) and MICA_v1 (rs2523497) variants, or HCP-5_v1 (rs2395029) was typical of low-responder HLA-Cw6^−^ or HLA-Cw6^+^ patients, respectively (Figure 1). 

Finally, SNPs in *NFKBIZ* (rs4683946, rs7637230), *LTA* (rs1799724) and *TNFA* (rs1800610) were found indistinctly in HLA-Cw6^+^ and HLA-Cw6^−^ patients, even though SNPs in *LTA* or *TNFA* and *NFKBIZ* characterized low- and high-responder patients, respectively (Figure 1). An analysis of SNP patterns performed by CAP on patient populations clustered based on their clinical response rate to ustekinumab (responders up to 2-year treatment, non-responders or only partially responders for primary failure or responders loosing responsiveness over time for secondary failure) (Figure S1), showed that the 59.6% of patients efficaciously responding to ustekinumab is characterized by the same SNPs, in particular SNPs of *HLA-C* (rs12191877, rs10484554, rs12189871 and rs4406273), HLA-C promoter (rs13191343 and rs13207315), *LCE* (rs6701730 and rs12030223), *PSORS1C3* (rs1265181) and *CCHCR1* (rs130076 and rs746647) genes, typically also found in the high-responder HLA-Cw6^+^ patients (Figure 1).

Two out of 17 of patients lost responsiveness to ustekinumab over time, and 8 out of 21 non-responders for primary failure of the drug were found in the group of responders (Figure S1). The non-responder group for primary failure was randomly distributed in the CAP plot, whereas more than 50% of non-responders for secondary failure mainly clustered in the left upper and lower panels of the graph (Figure S1) and were characterized by a SNP pattern similar to that found in low-responder patients shown in Figure 1. In parallel, CAP performed on non-responder patient population, grouped based on their BMI and presence/absence of HLA-Cw6 showed that a BMI >30 determined a failure of response to ustekinumab, especially in HLA-Cw6^−^ patients (11 out of 17 subjects, 64.7%). Consistently, the absence of HLA-Cw6 allele was also typical of non-responder population with a BMI <30 (14 out of 17 subjects, 82.4%) (Figure S2), confirming the finding that HLA-Cw6 allele predispose to responsiveness to ustekinumab [27]. However, BMI >30 determined a failure of response to ustekinumab also in non-responder patients carrying out HLA-Cw6 and associated alleles (rs12189871, rs4406273, rs12191877, rs10484554) (Figure S2).

### 3.2. Optimal Response to Ustekinumab Associates with the Allelic Variant Status of SNPs in the Intergenic Region Upstream of HLA-C, as Well as in PSORS1C3 and MICA Genes

**Figure 2:**
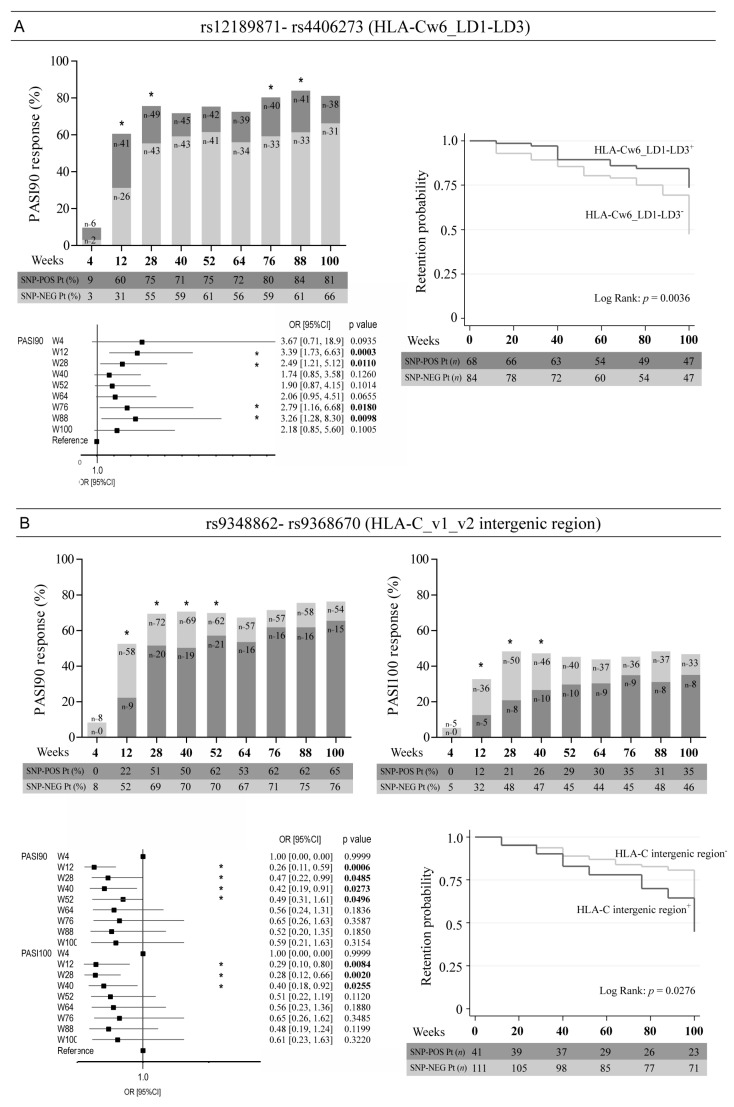
Association analysis between rs12189871, rs4406273, rs9348862 and rs9368670 SNPs in HLA-C intergenic region and clinical responses to ustekinumab treatment. Univariate logistic regression analysis was performed to evaluate the association between (**A**) rs12189871 (HLA-Cw6_LD1) or rs4406273 (HLA-Cw6_LD3) and (**B**) rs9348862 (HLA-C_v1 intergenic region) or rs9368670 (HLA-C_v2 intergenic region) with the clinical response to ustekinumab after 4, 12, 28, 40, 52, 64, 76, 88 and 100 weeks of treatment, in a cohort of patients (*n* = 152) affected by moderate-to-severe psoriasis. Graphs show the percentage of patients carrying (dark grey bars) or not (light grey) the SNPs and achieving 90% reduction of PASI (PASI90) (**A**,**B**) and 100% reduction (PASI100) (**B**). Rs12189871, rs4406273 (**A**) and rs1131118 (HLA-Cw6) (not shown), as well as rs9348862 and rs9368670 (**B**) showed the same pattern of presence/absence in the psoriatic population and, thus, identical logistic regression curves. Numbers in the bars indicate the exact number of subjects carrying or not the alleles. The tables under the graphs show the percentage of patients (Pt) carrying (SNP-POS) or not carrying (SNP-NEG) the relevant alleles. * *p* values < 0.05 were considered significant. The univariate analysis is also summarized in the forest plots (**A** and **B**). The condition of good response to ustekinumab is more likely to occur in the group of patients carrying (**A**) or not carrying (**B**) the allele, as indicated by the odds ratio (OR) >1 and <1, respectively. Squares on the *x*-axis shows OR estimates for each observation point and the error bars represent 95% confidence interval (CI). * *p* values < 0.05 were considered significant and indicated in bold font. Graphs of comparison between retention probability curves of HLA-Cw6_LD1-LCD3^+^ vs. HLA-Cw6_LD1-LD3^-^ (**A**) or HLA-C^+^ vs. HLA-C^-^ intergenic region (**B**) subgroups are shown. The tables under the graphs show the number of patients (Pt) carrying (SNP-POS) or not carrying (SNP-NEG) the relevant alleles. Retention probability was calculated using the log rank test. * *p* values < 0.05 were considered significant.

We next evaluated the influence of single genetic variants of the SNP panel used for NGS analysis on the clinical response of patients to ustekinumab at 4–100 weeks of treatment. To this end, the univariate logistic regression analysis between the SNP status in patients and the clinical response, expressed as PASI75, PASI90 and PASI100 achievement, was carried out. We identified four SNPs in the intergenic region (~12–26 kb) upstream of HLA-*C* associated with an optimal response to ustekinumab (Figure 2). 

In particular, patients carrying rs12189871 (HLA-Cw6_LD1) or rs4406273 (HLA-Cw6_LD3), present respectively at −12,055 and −26,000 bp of HLA-C gene, more successfully achieved PASI90 endpoint at week 12 (OR = 3.39, 95% CI: 1.73–6.63, *p* = 0.0003), week 28 (OR = 2.49, 95% CI: 1.21–5.12, *p* = 0.0110), week 76 (OR = 2.79, 95% CI: 1.16–6.68, *p* = 0.0180) and at week 88 (OR = 3.26, 95% CI: 1.28–8.30, *p* = 0.0098) (Figure 2A). Interestingly, both SNPs showed identical regression curves and co-segregated with HLA-Cw6 allele, carrying HLA-Cw6^+^ patients both rs12189871 and rs4406273 SNPs. Similarly, for both rs9348862 and rs9368670 (HLA-C intergenic region), located at −12,878 and −13,013 bp of *HLA-C*, we found identical regression curves, even though their presence negatively correlated with response to ustekinumab (Figure 2B). In fact, patients with wild-type alleles showed a better response to the drug compared with subjects carrying rs9348862 and rs9368670 SNPs, especially at early time-points of treatments (for PASI90 achievement at week 12, OR = 0.26, 95% CI: 0.11–0.59, *p* = 0.0006; for PASI100 achievement at week 12, OR = 0.29, 95% CI: 0.10–0.80, *p* = 0.0084). 

The probability of continuing the treatment with ustekinumab over time was significantly higher in patients carrying rs12189871 and rs4406273 (Figure 2A) and lower in patients with rs9348862 and rs9368670 SNPs in HLA-C intergenic region, as reported in the drug survival graphs shown in Figure 2B.

**Figure 3:**
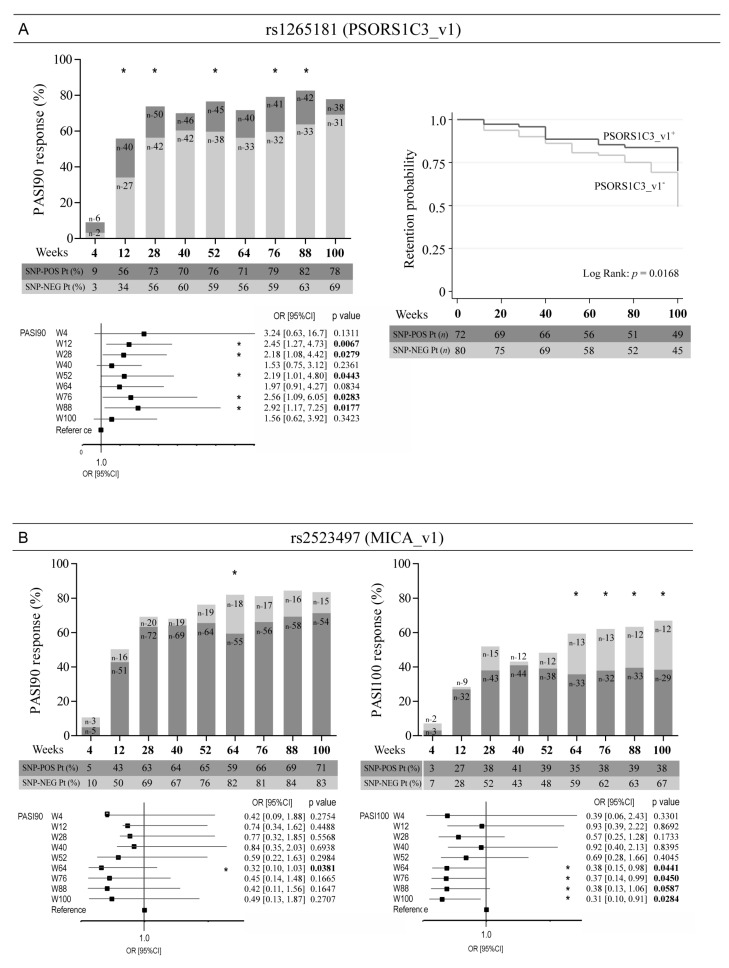
Association analysis between rs1265181 in *PSORS1C3* and rs2523497 in *MICA* and clinical response to ustekinumab. Rs1265181 (PSORS1C3_v1)-positive (dark grey bars) and rs2523497-negative patients (light grey bars) reached a significant better response to ustekinumab than negative (light grey bars) (**A**) or positive (dark grey bars) (**B**) patients, respectively, in terms of achievement of PASI90 (**A**), or PASI90 and PASI100 (**B**), as evaluated by logistic regression analysis. Numbers in the bars indicate the exact number of subjects carrying or not the alleles. The tables under the graphs show the percentage of patients (Pt) at the time-points of observation, carrying (SNP-POS) or not carrying (SNP-NEG) the relevant alleles. * *p* values < 0.05 were considered significant. The univariate logistic regression analyses are also summarized in forest plots (**A**,**B**). The condition of good response to ustekinumab is more likely to occur in the group of patients carrying the (**A**) or not carrying the (**B**) allele, as indicated by the odds ratio (OR) > 1 and < 1, respectively. Squares on the *x*-axis shows OR estimates for each observation point and the error bars represent 95% CI. * *p* values < 0.05 were considered significant and indicated in bold font. In (**A**), the graph of comparison between retention probability curves of PSORS1C3_v1^+^ vs. PSORS1C3_v1^−^ subgroups is shown. * *p* values < 0.05 were considered significant.

Other SNPs relevant for the response to ustekinumab included rs1265181 present in the promoter region of *PSORS1C3* and rs2523497 in the first intron of *MICA* (Figure 3). In particular, the presence of rs1265181 and the absence of rs2523497 SNP determined a better response to ustekinumab at most time-points of treatment and a significant prolonged time of treatment in the psoriatic population (Figure 3 and data not shown). Of note, rs2523497 absence in *MICA* gene resulted in the achievement of PASI100 response by patients, especially at late time-points of treatment with ustekinumab (week 88, OR = 0.38, 95% CI: 0.13–1.06, *p* = 0.05; week 100, OR = 0.31, 95% CI: 0.10–0.91, *p* = 0.0284) (Figure 3B).

### 3.3. Allelic Variants of Genes Involved in Epidermal Homeostasis and Barrier Function and TNFA Affect the Clinical Response to Ustekinumab

**Figure 4:**
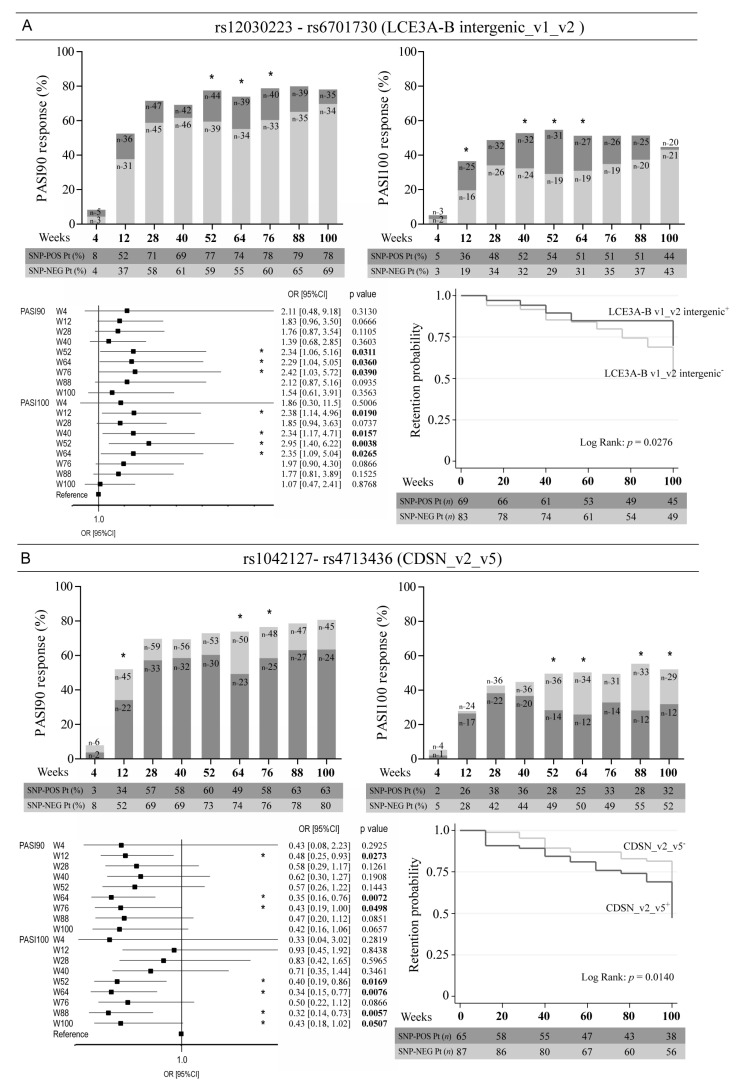

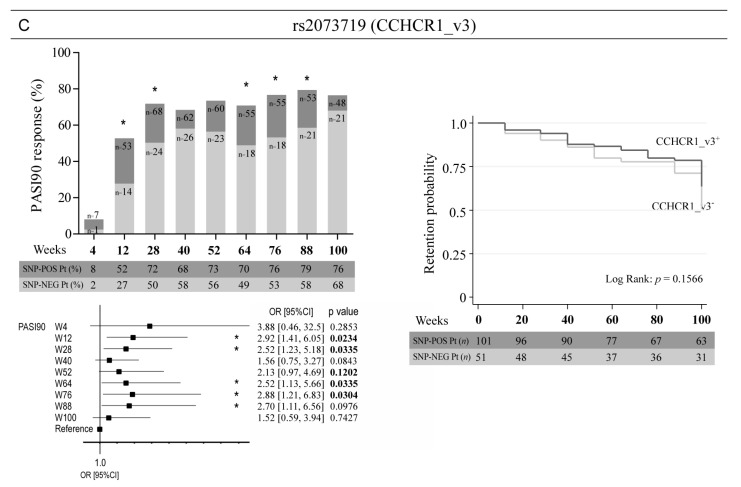
Association analysis between clinical response to ustekinumab and SNPs in *LCE3A-B* (rs12030223, rs6701730), *CDSN* (rs1042127, rs4713436) and *CCHCR1* (rs2073719). (**A**) Patients carrying out rs12030223 or rs6701730 SNPs in *LCE3A-B* (LCE3A-B v1_v2) (dark grey bars) showed a better response to ustekinumab than SNP-negative patients (light grey bars), with the achievement of PASI90 and PASI100 at the indicated time-points of observation. (**B**) *Vice versa* the absence of the rs1042127 (CDSN_v2) and rs4713436 (CDSN_v5) (light grey bars) determined PASI90 and PASI100 achievement, as compared with SNP-positive patients (dark grey bars). (**C**) Rs2073719 (CCHCR1_v3)-positive patients (dark grey bars) reached a significant better response to ustekinumab than SNP-negative subjects (light grey bars). Rs12030223 and rs6701730 (**A**) as well as rs1042127 and rs4713436 (**B**) showed the same pattern of presence/absence in the psoriatic population and, thus, identical logistic regression curves. Numbers in the bars indicate the exact number of subjects carrying or not the alleles. The tables under the graphs show the percentage of patients (Pt) carrying (SNP-POS) or not carrying (SNP-NEG) the relevant alleles. * *p* values < 0.05 were considered significant. Regression analyses are also summarized in forest plots (**A**–**C**). Squares on the *x*-axis shows OR estimates for each observation point and the error bars represent 95% CI. * *p* values < 0.05 were considered significant and indicated in bold font. Graphs of comparison between retention probability curves of rs12030223/rs6701730 (**A**), rs1042127/ rs4713436 (**B**) and rs2073719 (**C**) are shown. The tables under the graphs show the number of patients (Pt) carrying (SNP-POS) or not carrying (SNP-NEG) the relevant alleles. * *p* values < 0.05 were considered significant.

Univariate regression analyses also revealed significant associations between response to ustekinumab and SNPs in genes encoding proteins involved in keratinocyte proliferation and terminal differentiation. In particular, two co-segregating SNPs, namely rs12030223 and rs6701730, located in the intergenic region between *LCE3B* and *LCE3A* were found to associate to an optimal drug outcome (Figure 4A). In fact, psoriatic patients carrying rs12030223 and rs6701730 more successfully achieved PASI90 endpoint at week 52 (OR = 2.34, 95% CI: 1.06–5.16, *p* = 0.0311), week 64 (OR = 2.29, 95% CI: 1.04–5.05, *p* = 0.0360) and week 76 (OR = 2.42, 95% CI: 1.03–5.72, *p* = 0.0390), and PASI100 at weeks12 (OR = 2.38, 95% CI: 1.14–4.96, *p* = 0.0190), week 40 (OR = 2.34, 95% CI: 1.17–4.71, *p* = 0.0157), week 52 (OR = 2.95, 95% CI: 1.40–6.22, *p* = 0.0038) and at week 64 (OR = 2.35, 95% CI: 1.09–5.04, *p* = 0.0265) (Figure 4A). For both SNPs, we observed the same pattern of presence in the psoriatic population and, thus, identical regression curves. In these patients, the probability to continue the therapy with ustekinumab over time was significantly higher as compared with patients carrying wild-type alleles (Figure 4A). Other two SNPs, rs1042127 and rs4713436, present in exon 2 of *CDSN* overlapping with intron 1 of *PSORS1C1* at Chr6:31084170 and Chr6:31084639 positions, respectively, were found to co-segregate in all patients and to negatively influence the response to ustekinumab. In this case, patients with wild-type alleles of *CDSN* responded better to ustekinumab in terms of achievement of PASI90 (week 76, OR = 0.43, 95% CI: 0.19–1.00, *p* = 0.0498) and PASI100 up to week 100 (OR = 0.43, 95% CI: 0.18–1.02, *p* = 0.0507) and showed higher probability to continue drug treatment over time as compared with patients carrying rs104127 and rs4713436 SNPs (Figure 4B). 

Among the analysed SNPs in *CCHCR1*, rs2073719 (CCHCR1_v3) significantly resulted associated to optimal response to ustekinumab at weeks 12, 28, 64, 76 and 88 (OR = 2.70, 95% CI: 1.11–6.56, *p* = 0.0976), even though differences in drug retention probability of patients carrying or not rs2073719 did not result statistically significant (Figure 4C).

**Figure 5:**
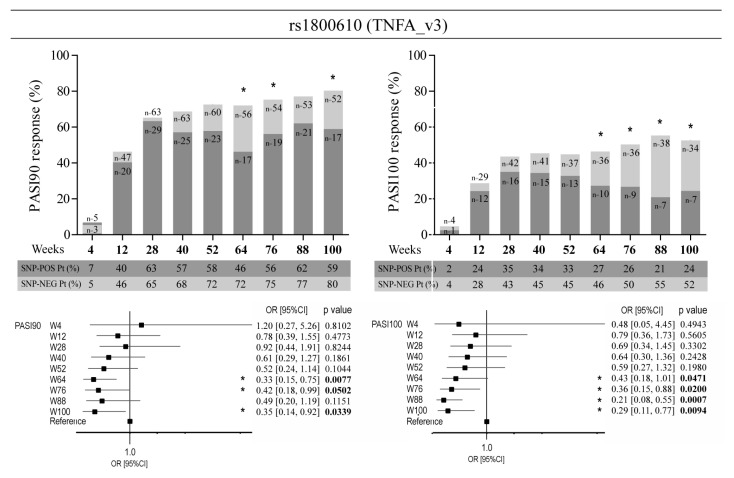
Association analysis between rs1800610 in *TNFA* and response to ustekinumab. Rs1800610 (TNFA_v3)-positive patients (light grey bars) reached a significant better response to ustekinumab than SNP-negative (dark grey bars) subjects, in terms of achievement of PASI90 and PASI100, as evaluated by logistic regression analysis. Numbers in the bars indicate the exact number of subjects carrying or not the alleles. The tables under the graphs show the percentage of patients (Pt) at the time-points of observation, carrying (SNP-POS) or not carrying (SNP-NEG) the relevant alleles. * *p* values < 0.05 were considered significant. The univariate logistic regression analyses are also summarized in forest plots. The condition of good response to ustekinumab is more likely to occur in the group of patients not carrying the allele, as indicated by the OR < 1. Squares on the *x*-axis shows OR estimates for each observation point and the error bars represent 95% CI. * *p* values < 0.05 were considered significant and indicated in bold font.

The absence of rs1800610 SNP in intron 1 of *TNFA* (TNFA_v3) determined an optimal response to ustekinumab of patients, who achieved PASI90 and PASI100 at 64–100 weeks of treatment more frequently compared with patients carrying the polymorphism (for PASI90 achievement at week 100, OR = 0.35, 95% CI: 0.14–0.92, *p* = 0.0339; for PASI100 achievement at week 100, OR = 0.29, 95% CI: 0.11–0.77, *p* = 0.0094) (Figure 5).

Finally, multivariate logistic regression analysis did not reveal significant any associations between the SNPs relevant for response to ustekinumab and other variables, such as clinical, demographic and anthropometric characteristics of patients, possibly due to the reduced population size. 

## 4. Discussion

The developmental strategy of therapeutics for psoriasis, in particular biologics, has been mainly based on the targeting of pathogenic cytokines and cellular signaling pathways, without taking into consideration the potential influence of the genetic backgrounds of patients and, thus, their intrinsic/individual ability to respond to the drug. Ustekinumab was conceived and employed starting from 2009 for the treatment of psoriasis, specifically for the inhibition of the drivers of Th1/Tc1 and Th17/Tc17 responses, with consequent reductions in IFN-γ- and IL-17-dependent systemic and cutaneous inflammation [2]. Although ustekinumab shows sustained drug survival in the psoriatic population, not all candidate patients can achieve valuable and long-term clinical improvement [21].

In the past, pharmacogenomic studies aimed at evaluating the influence of allelic variants of psoriasis susceptibility genes on the clinical response to ustekinumab identified some SNPs associated with drug outcome. They included SNPs of HLA-C, namely HLA-Cw6 [27,28], *IL17F* (rs763780) [29], *TNFRSF1B* (rs1061622) [30], *IL12B* (rs6887695) [31], *IL1B* (rs1143623, rs1143627), *TIRAP* (rs8177374), *TLR5* (rs5744174) [32], *CHUK* (rs11591741), *C17orf51* (rs1975974), *ZNF816A* (rs9304742), *STAT4* (s7574865), *SLC22A4* (rs1050152), *Corf72* (rs774359) [33] and *ERAP1* (rs151823 and rs26653) [34]. A recent genome-wide study also identified a novel SNP (rs35569429) potentially associated with response to ustekinumab [35]. All these SNPs were studied in different cohorts of patients with different ethnicities who underwent ustekinumab treatment for different time periods, most of them in combination with other two or three SNPs. Only few studies analyzed sets of polymorphisms and identified a group of allelic variants associated with response to ustekinumab [32,33]. 

Our pharmacogenomic study extends these previous observations of the influence of allele variants on the clinical outcomes of ustekinumab. For the first time, more than 400 genetic variants were analyzed simultaneously by using massive DNA sequencing, looking for SNPs potentially predicting the clinical response to ustekinumab of a Caucasian population of *n* = 152 patients affected by plaque-type psoriasis who underwent long-term follow-up evaluations (up to 2 years of treatment). 

Our analysis led to the identification of a set of psoriasis-risk SNPs and haplotypes significantly associated with an excellent response to ustekinumab. Among them, the allelic status of four SNPs (rs12189871, rs4406273, rs9348862 and rs9368670), all present in a region located between −12,055 and 26,000 bp upstream of *HLA-C*, strongly influenced the response to ustekinumab. The optimal drug outcome and retention probability depended on the presence of rs12189871 and rs4406273 and absence of rs9348862 and rs9368670 SNPs in patients who more successfully achieved PASI90 and PASI100. Of note, the presence of HLA-Cw6 polymorphism (rs1131118) in exon 3 of *HLA-C* and rs12189871 (HLA-Cw6_LD1) or rs4406273 (HLA-Cw6_LD3) in HLA-C intergenic region was identical in all psoriatic patients, indicating that these allelic variants co-segregate, even though they are distant from each other. Thus, our findings indirectly confirm our previous studies demonstrating the importance of HLA-Cw6 in conferring responsiveness to ustekinumab in patients carrying this allele [23]. Similarly, HLA-Cw6 allele and rs12189871 or rs4406273 SNPs were previously found to be associated with the clinical response to secukinumab [19]. The reason HLA-Cw6 allele associates with response to biologics blocking IFN-γ and IL-17 cytokines could depend on its capability to influence the presentation of epitopes of different autoantigens, such as the cathelecidin LL37, which is efficiently recognized by circulating CD8^+^ T cells with a psoriasis-like cytokine profile (IFN-γ^high^ and IL-17^high^) [15]. On the other hand, SNPs present in the region upstream of HLA-C up to −26,000 bp, which contain genomic regions potentially transcribing long noncoding RNA (i.e., LINC02571 and LOC112267902), could influence the expression levels of HLA-C class I molecules in immune and resident skin cells, and, consequently, the expansion rate of CD8^+^ IFN-γ- and IL-17-producing T cells. Future studies exploring the functional role of specific HLA-C alleles in IFN-γ/IL-17-dependent immune responses will be fundamental not only to unveil their pathogenic activity in psoriasis but also to understand why specific HLA-C haplotypes effectively respond to the ustekinumab treatment.

A significant association was also observed for rs1265181 present at –1691 bp from the transcription initiation site of *PSORS1C3*, a lncRNA discovered in a linkage analysis on psoriasis and immune-mediated diseases [36], regulating the expression of the nearby POU5F1 gene, which encodes OCT4, the main regulator of pluripotency in stem cells [37]. *PSORS1C3* has two endogenously active promoters and two sets of transcripts, short and long variants, differently regulating OCT4 transcripts [37]. The presence of rs1265181 SNP in the promoter region of *PSORS1C3* could indirectly upregulate OCT4 levels and thus enhance stemness in skin cells. On the other hand, IFN-γ and/or IL-17 could regulate *PSORS1C3* promoter via activation/repression of transcription factors binding the region where rs1265181 SNP is located. 

Other SNPs relevant for the response to ustekinumab included rs2523497 and rs1800610 potentially located present in the first intron of *MICA* and *TNFA* genes, respectively. Although both *MICA* and *TNFA* genes have a high degree of polymorphism, and their allelic diversity has been reported in association with some autoimmune diseases, including psoriasis [29] and with response to anti-TNF treatment [38], rs2523497 and rs1800610 have never been associated with psoriasis condition. Only rs1800610 in *TNFA* has been correlated with chronic diseases, infections, and cancer susceptibility [39,40]. Indeed, we found that the absence of rs2523497 and rs1800610, rather than the presence, was determinant for optimal drug outcome, especially at late time-points of treatment with ustekinumab. Studies of linkage analysis will be necessary to unveil associations of rs2523497 and rs1800610 with psoriasis susceptibility.

Moreover, we found interesting associations between clinical response to ustekinumab and allelic variants of genes predisposing to psoriasis and influencing epidermal homeostasis and barrier function. We identified rs12030223 and rs6701730, two co-segregating SNPs located in LCE gene cluster, rs1042127 and rs4713436 in exon 2 of *CDSN* overlapping with *PSORS1C1* and rs2073719 in *CCHCR1*. While SNPs in *LCE* and *CCHCR1* predispose to an optimal response to ustekinumab, SNPs in *CDSN* reduced drug efficacy in patients. All these allelic variants are likely involved in the pathogenic responses induced by IFN-γ and/or IL-17 in psoriatic keratinocytes, in terms of terminal differentiation and proliferation, two processes contributing to epidermal acanthosis typical of psoriatic lesions [2]. In particular, LCE genes, located in the epidermal differentiation complex of *PSORS4* locus, encode structural proteins with a role in epithelial barrier formation, as well as peptides with antimicrobial activity [41]. Previous studies identified a number of conserved, non-coding elements within LCE intergenic region exhibiting dynamic regulatory activity and coordinating LCE expression in differentiating or proliferating cells [42]. Rs12030223 and rs6701730 SNPs are located between *LCE3B* and *LCE3A*, in an intergenic region potentially involved in the regulation of expression levels of LCE3 genes. This genomic sequence could have regulatory functions similar to the entire LCE3B/C region, whose deletion leads to increased LCE3A mRNA expression in psoriatic skin under the influence of Th1 and Th17 cytokines [43]. In parallel, we observed a strong reduction of ustekinumab efficacy in patients carrying rs1042127 and rs4713436, two SNPs distant about 500 bp from each other, in a genomic region transcribing both CDSN and PSORS1C1 mRNAs. While rs1042127 can give rise to a missense variant of *CDSN* strongly impacting on corneocyte adhesion and skin desquamation, rs4713436 SNP has no apparent effects, as it determines a synonymous amino acid substitution in CDSN. It is likely that patients with more severe forms of psoriasis respond to treatments less efficiently and, therefore, only subjects carrying out wild-type alleles of *CDSN*, and not variants correlating with disease severity, can substantially improve their condition after ustekinumab therapy. Indeed, rs1042127 and rs4713436 SNPs are located in exon 2 of *CDSN* where several SNPs with functional effects distribute. They include rs1062470 not investigated in this study, a SNP located at 265 bp and 204 bp from rs1042127 and rs4713436 SNPs, respectively, which has been found to associate with increased risk of psoriasis severity [44]. The potential effects of rs1042127 and rs4713436 SNPs on PSORS1C1 expression and function in patients undergone ustekinumab therapy is unpredictable. Finally, among the SNPs analysed in *CCHCR1*, rs2073719 only resulted significantly associated with optimal response to ustekinumab. Due to its position in intron 13 of *CCHCR1*, 18 bp from exon 13, this SNP is irrelevant for amino acid composition of CCHCR1 protein, even if its potential regulatory function of CCHCR1 mRNA cannot be excluded. In fact, several CCHCR1 mRNA and protein variants can be produced depending on SNP presence and haplotypes [45]. CCHCR1 influences keratinocyte proliferation by regulating cytoskeleton as well as other processes including RNA surveillance and transport [46]. The function of CCHCR1 isoforms in psoriasis together with the IFN-γ and IL-17-dependent mechanisms regulating their expression pattern in psoriatic skin remains to be elucidated.

Coherently with univariate regression analysis, we identified specific patterns of psoriasis-risk SNPs significantly associated with optimal response to ustekinumab in patient populations stratified for HLA-Cw6. CAP demonstrated that HLA-Cw6^+^ patients highly responding to the drug were characterized by the co-presence of allelic variants of LCE3A-B intergenic region (rs12030223, rs6701730), *CDSN* (rs33941312), *CCHCR1* (rs2073719, rs746647 and rs130076), *PSORS1C3* (rs1265181), HLA-C intergenic region (rs12189871, rs4406273, rs12191877, rs10484554), HLA-C promoter region (rs13207315, rs13191343) and *HCP5* (rs2395029). A similar pattern of SNPs was also found by performing CAP on patient populations clustered based on their clinical response rate to ustekinumab. Most of them corresponded to SNPs found significantly associated to drug response by univariate analysis, with the addition of other allelic variants of the same genomic regions. On the other hand, the presence of SNPs in *TNFSF15* (rs3810936, rs6478108, rs4263839, rs6478109), *CCHCR1* (rs375143475) *CDSN* (rs1042127, rs4713436), *MICA* (rs2523497) and in genomic region upstream of HLA-C (rs116350468, rs115727572, rs17192533) characterized HLA-Cw6^−^ patients. Concerning SNPs typical of HLA-Cw6^−^ patients, those present in *MICA* (rs2523497) and *CDSN* (rs1042127, rs4713436) were typical of the group showing moderate response to the drug, accordingly to the findings that its absence determined a better response to ustekinumab in regression models. CAP also identified SNPs which were present indistinctly in HLA-Cw6^+^ and HLA-Cw6^−^ patients. Among them the absence of rs1800610 SNP in *TNFA* determined the achievement of PASI100 at 64–100 weeks of treatment with ustekinumab. Interestingly, HLA-C variants characterizing HLA-Cw6^+^ and HLA-Cw6^−^ patients were different and mutually exclusive, indicating that the allelic status of HLA-C genic and intergenic region strongly influences the drug outcome, even in absence of HLA-Cw6 allele. Consistently with these findings, we previously showed that eight SNPs in HLA-C and upstream region (rs13207315, rs6900444, rs12189871, rs12191877, rs4406273, and rs10484554), including HLA-Cw6 (rs1131118), associated with an excellent response to the IL-17A blocker secukinumab [19]. In addition, patients highly responding to secukinumab carried out specific and distinct patterns of SNPs in HLA-C genomic region depending on HLA-Cw6 allele status.

Concomitant to HLA-Cw6 and associated haplotypes, BMI is also fundamental for an optimal response to ustekinumab. In fact, a BMI >30 could determine a failure of response in both HLA-Cw6^+^ and HLA-Cw6^−^ patients, even though most of the of non-responders were HLA-Cw6^−^. Consistently, the absence of HLA-Cw6 allele was also typical of the non-responder population with a BMI <30, confirming the findings that HLA-Cw6 allele together with the other allelic variants predispose to responsiveness to ustekinumab. These findings extend our previous studies showing that HLA-Cw6 positivity and BMI <30 kg/m^2^ are important predictors of response to ustekinumab [21].

Taken together, our data support the idea that HLA-Cw6 status efficiently stratifies psoriasis patients in terms of response to ustekinumab, showing HLA-Cw6^+^ patients a specific psoriasis-risk SNP pattern associated with an optimal response to the drug. Most of these SNPs were predominantly located in genes of *PSORS1*, indicating the relevance of this locus in the predisposition of all patients to psoriasis and in the drug outcome.

Ustekinumab is the foremost biologic evaluated for efficacy in patients [47], and pharmacogenetic studies have identified genetic biomarkers of response to the drug [48,49]. Our study extends the list of SNPs potentially predictive of efficacy of ustekinumab, useful to increase the success rate of the therapy, also by guaranteeing long-term responses in patients. From 2018, ustekinumab had clinically actionable pharmacogenomic tags that would require testing of biomarkers before treatment [50]. Among them, FDA approved *IL12A*, *IL12B*, *IL23A* as single biomarkers (https://www.fda.gov/drugs/science-and-research-drugs/table-pharmacogenomic-biomarkers-drug-labeling, (accessed on 8 November 2022)). However, given the polygenicity, clinical heterogeneity and immunological complexity of psoriasis, evaluations of a multi-gene marker/SNP panels will be more appropriate to support the right clinical decision.

Finally, although more effective biologics targeting IL-17 or IL-23p19 have been introduced into the market, the interest on ustekinumab is increasing due the availability of several ustekinumab biosimilars in the very next future [51]. Ustekinumab biosimilars will be commercialized at significantly lower price broadening dramatically the number of patients who may benefit from this drug as had occurred for the anti-TNF biosimilars [52]. Thus, the identification of potential predictors of optimal response to ustekinumab could further optimize the access in the clinical practice of anti-IL-12/IL-23 biosimilars.

## Figures and Tables

**Table 1:** Demographic and disease characteristics of patients (*n* =152).

Characteristics	
Male/female (n)	98/54
	*Mean ± SD (range)*
Age (years)	50.6 ± 14.4 (18–85)
PASI at baseline	19.1 ± 10.8 (5–62)
PASI at follow-up (n)	
Week 4 (137)	10.2 ± 7.83 (0–45)
Week 12 (152)	5.23 ± 6.97 (0–40)
Week 28 (143)	3.35 ± 5.59 (0–40)
Week 40 (136)	3.01 ± 5.23 (0–40)
Week 52 (123)	2.39 ± 3.71 (0–18)
Week 64 (115)	2.56 ± 3.89 (0–20)
Week 76 (106)	2.31 ± 3.91 (0–25)
Week 88 (103)	2.18 ± 3.54 (0–18)
Week 100 (94)	1.93 ± 3.27 (0–18)
Duration of disease (years)	21.1 ± 13.4 (1–67)
Age at disease onset (years)	27.1 ± 13.7 (7–73)
Weight (kg)	81.2 ± 20.9 (23–168)
BMI (kg/m^2^)	29.1 ± 17.8 (15.2–40.4)
*Biologics before anti-IL-12/IL-23 therapy*	*n* (*%*)
0 prior biologics	85 (55.2)
1 prior biologics	38 (24.7)
2 prior biologics	22 (14.3)
≥3 prior biologics	9 (5.8)
*Comorbidities*	98
Hypertension	42
Type 2 diabetes mellitus	7
Hyperlipidemia	19
Depression	3
Obesity (BMI ≥ 30.0–34.9 kg/m^2^)	27

## Data Availability

Databases containing patient’s information can be found at Dept. of Systems Medicine, “Tor Vergata” University of Rome (marco.galluzzo@uniroma2.it) whereas databases containing SNP data supporting reported results can be found at IDI-IRCCS (c.albanesi@idi.it).

## Supplementary Materials

The following are available online at https://www.mdpi.com/article/10.3390/vaccines10111977/s1, Figure S1: SNPs patterns in patient populations clustered based on their clinical response rate to usteki-numab. Figure S2: SNPs patterns in patient populations clustered based on BMI values and HLA-Cw6 allele presence/absence. Table S1: List of the analyzed SNPs.
