# Supplementary material for: Allelic Variants of HLA-C Upstream Region, PSORS1C3, MICA, TNFA and Genes Involved in Epidermal Homeostasis and Barrier Function Influence the Clinical Response to Anti-IL-12/IL-23 Treatment of Patients with Psoriasis
Source: Vaccines (Basel). 2022 Nov 21;10(11):1977. doi: 10.3390/vaccines10111977 (PMC9695538; doi:10.3390/vaccines10111977)
Supplement: Supplementary file 1 [file vaccines-10-01977-s001.zip › vaccines-2028504-supplementary.pdf]

❖ **Supplementary Files**

Supplementary Table S1. List of the analyzed SNPs

Genome position	Gene	REF/ALT	SNP name	dbSNP ID	MAF
**Antigen presentation**					
Chr5:96101822	ERAP1	C/T	ERAP1_v7	rs61733809	0.0037
Chr5:96101907	ERAP1	C/T	ERAP1_v8	rs536860924	0.0004
Chr5:96101944	ERAP1	A/G	ERAP1_v1	rs27524	0.6296
Chr5:96101959	ERAP1	C/T	ERAP1_v2	rs27525	0.4532
Chr5:96101993	ERAP1	C/T	ERAP1_v9	rs73138691	0.0085
Chr5:96118811	ERAP1	G/A	ERAP1_v10	rs146423238	0.0046
Chr5:96118852	ERAP1	G/C	ERAP1_v3	rs27044	0.7158
Chr5:96118866	ERAP1	C/T	ERAP1	rs17482078	0.1837
Chr5:96119028	ERAP1	G/A	ERAP1_v11	rs182760682	0.0029
Chr5:96119044	ERAP1	T/A	ERAP1_v12	rs62364748	0.0781
Chr5:96119055	ERAP1	T/C	ERAP1_v13	rs11738810	0.1061
Chr5:96124296	ERAP1	C/G	ERAP1_v14	rs2278017	0.0007
Chr5:96124330	ERAP1	T/C	ERAP1_v4	rs30187	0.6491
Chr5:96124373	ERAP1	C/T	ERAP1_v15	rs78649652	0.0093
Chr5:96124447	ERAP1	G/C	ERAP1_v5	rs30186	0.7886
Chr5:96124453	ERAP1	A/G	ERAP1_v6	rs11743410	0.1245
Chr5:96124534	ERAP1	G/A	ERAP1_v16	rs112124378	0.0017
Chr5:96124687	ERAP1	A/G	ERAP1_v17	rs113202554	0.0038
Chr5:96139034	ERAP1	C/T	ERAP1_v18	rs73148306	0.0277
Chr5:96139061	ERAP1	G/A	ERAP1_v19	rs200264416	0.0031
Chr5:96139066	ERAP1	C/T	ERAP1_v20	rs10062964	0.1724
Chr5:96139250	ERAP1	C/G	ERAP1_v21	rs26653	0.2250
Chr6:31155532	PSORS1C3	A/C	PSORS1C3_v2	rs28360057	0.1503
Chr6:31155539	PSORS1C3	T/G	PSORS1C3_v3	rs4713443	0.3849
Chr6:31155548	PSORS1C3	A/G	PSORS1C3_v4	rs4713444	0.4315
Chr6:31155615	PSORS1C3	A/G	PSORS1C3_v5	rs28360058	0.1458
Chr6:31155659	PSORS1C3	G/A	PSORS1C3_v6	rs9263845	0.1594
Chr6:31155670	PSORS1C3	G/A	PSORS1C3_v7	rs9263846	0.1781
Chr6:31155785	PSORS1C3	G/C	**PSORS1C3_v1**	rs1265181	0.2115
Chr6:31155790	PSORS1C3 region	C/T	PSORS1C3_v8	rs116116256	0.0241
Chr6:31155803	PSORS1C3 region	C/T	PSORS1C3_v9	rs9405015	0.0155
Chr6:31236622	HLA-C	C/A	HLA-C_v1	rs1130538	0.0000
Chr6:31236639	HLA-C	G/C	HLA-C_v2	rs1071643	0.0364
Chr6:31236643	HLA-C	T/C	HLA-C_v3	rs1130552	0.0170
Chr6:31236668	HLA-C	T/C	HLA-C_v4	rs1130576	0.0274
Chr6:31236679	HLA-C	T/C	HLA-C_v5	rs1130580	0.3358
Chr6:31236690	HLA-C	T/G	HLA-C_v6	rs1130592	0.3189
Chr6:31236722	HLA-C	C/T	HLA-C_v7	rs1094	0.3231
Chr6:31236767	HLA-C	G/A	HLA-C_v8	rs1049579	0.0252
Chr6:31236800	HLA-C	G/A	HLA-C_v9	rs116229144	0.0179
Chr6:31236808	HLA-C	C/G	HLA-C_v10	rs1049650	0.0635
Chr6:31236813	HLA-C	C/A	HLA-C_v11	rs1049663	0.0114
Chr6:31236821	HLA-C	C/T	HLA-C_v12	rs1049668	0.0191
Chr6:31236836	HLA-C	A/G	HLA-C_v13	rs41289069	0.0239
Chr6:31236853	HLA-C	GT/AT	HLA-C_v14	rs1065711	0.1314
Chr6:31236853	HLA-C	GT/AC	HLA-C_v15	rs386698953	MNV

Chr6:31239108	HLA-C	T/A	HLA-Cw6	rs1131118	0.3143
Chr6:31239506	HLA-C	C/G	HLA-C exon2	rs1050414	0.1201
Chr6:31239518	HLA-C	C/T	HLA-C_v16	rs1050420	0.0000
Chr6:31239543	HLA-C	C/T	HLA-C_v17	rs1050428	0.0996
Chr6:31239577	HLA-C	A/C	HLA-C_v18	rs707911	0.0000
Chr6:31239585	HLA-C	C/T	HLA-C_v19	rs1050437	0.1729
Chr6:31239593	HLA-C	T/C	HLA-C_v20	rs41542719	0.2407
Chr6:31239601	HLA-C	C/T	HLA-C_v21	rs281860337	0.0000
Chr6:31239614	HLA-C	G/A	HLA-C_v22	rs1050444	0.1573
Chr6:31239616	HLA-C	C/T	HLA-C_v23	rs1050445	0.1142
Chr6:31241077	HLA-C region	T/C	HLA-C_promoter_v4	rs35976302	0.1272
Chr6:31241092	HLA-C region	C/G	HLA-C_promoter_v5	rs2523599	0.3645
Chr6:31241109	HLA-C region	C/T	HLA-C_promoter1	rs13191343	0.1394
Chr6:31241116	HLA-C region	C/T	HLA-C_promoter_v6	rs28367582	0.0414
Chr6:31241127	HLA-C region	T/C	HLA-C_promoter2	rs13207315	0.1429
Chr6:31241182	HLA-C region	C/T	HLA-C_ promoter3	rs6900444	0.4937
Chr6:31241207	HLA-C region	C/T	HLA-C_promoter_v7	rs6900458	0.2140
Chr6:31241215	HLA-C region	A/G	HLA-C_promoter_v8	rs58019823	0.1239
Chr6:31241241	HLA-C region	C/T	HLA-C_promoter_v9	rs114183633	0.0272
Chr6:31241260	HLA-C region	T/C	HLA-C_promoter_v9	rs2524088	0.0000
Chr6:31241270	HLA-C region	G/A	HLA-C_promoter_v10	rs6900323	0.2205
Chr6:31241294	HLA-C region	C/G	HLA-C_promoter_v11	rs2524087	0.0413
Chr6:31241311	HLA-C region	A/G	HLA-C_promoter_v12	rs2524086	0.1476
Chr6:31241353	HLA-C region	G/T	HLA-C_promoter_v13	rs34090104	0.0642
Chr6:31241370	HLA-C region	C/T	HLA-C_promoter_v14	rs6923313	0.3589
Chr6:31251829	HLA-C region	A/T	HLA-C	rs764918802	0.0003
Chr6:31251895	HLA-C region	A/G	HLA-C	rs2524057	0.0862
Chr6:31251924	HLA-C region	C/T	**HLA-Cw6_LD1**	rs12189871	0.0904
Chr6:31251937	HLA-C region	C/T	HLA-C	rs114529772	0.0309
Chr6:31251954	HLA-C region	A/G	HLA-C	rs115182712	0.0488
Chr6:31251962	HLA-C region	C/T	HLA-C	rs114020623	0.0485
Chr6:31252019	HLA-C region	G/C	HLA-C	rs113629572	0.0472
Chr6:31252026	HLA-C region	A/C	HLA-C	rs114652772	0.0470
Chr6:31252031	HLA-C region	A/G	HLA-C	rs115699806	0.0488
Chr6:31252033	HLA-C region	C/T	HLA-C	rs114108198	0.0162
Chr6:31252044	HLA-C region	T/C	HLA-C	rs749596247	0.0002
Chr6:31252075	HLA-C region	C/A	HLA-C	rs73728774	0.0625
Chr6:31252747	HLA-C region	T/C	**HLA-C_v1 intergenic region**	rs9348862	0.0859
Chr6:31252882	HLA-C region	C/T	**HLA-C_v2 intergenic region**	rs9368670	0.0841
Chr6:31252893	HLA-C region	G/C	HLA-C	rs1946117	0.0984
Chr6:31252909	HLA-C region	G/C	HLA-C	rs117116032	0.0057
Chr6:31252925	HLA-C region	C/T	HLA-Cw6_LD2	rs12191877	0.13253
Chr6:31252951	HLA-C region	G/T	HLA-C_v2	rs116350468	0.0357
Chr6:31253034	HLA-C region	T/G	HLA-C_v3	rs115727572	0.0357
Chr6:31265940	HLA-C region	C/T	HLA-C	rs17192519	0.3085
Chr6:31265971	HLA-C region	G/T	HLA-C	rs17198867	0.0796
Chr6:31265989	HLA-C region	G/A	HLA-C	rs17198874	0.3079
Chr6:31265998	HLA-C region	C/T	HLA-C	rs17198881	0.0679
Chr6:31266006	HLA-C region	A/G	HLA-C	rs78514002	0.0136
Chr6:31266015	HLA-C region	A/G	HLA-C	rs17198888	0.1773
Chr6:31266033	HLA-C region	G/A	HLA-C	rs17198895	0.3056
Chr6:31266041	HLA-C region	T/C	HLA-C	rs17192526	0.4381
Chr6:31266085	HLA-C region	C/G	HLA-C_v4	rs17192533	0.1028

Chr6:31266090	HLA-C region	G/A	**HLA-Cw6_LD3**	rs4406273	0.0775
Chr6:31266106	HLA-C region	C/T	HLA-C	rs115533686	0.0154
Chr6:31266117	HLA-C region	A/C	HLA-C_v5	rs2524095	0.5689
Chr6:31266151	HLA-C region	G/T	HLA-C_v6	rs7761855	0.0633
Chr6:31266189	HLA-C region	A/T	HLA-C	rs17198909	0.0487
Chr6:31266189	HLA-C region	AA/TG	HLA-C	rs386698993	MNV
Chr6:31266190	HLA-C region	A/G	HLA-C_v7	rs2853922	0.6029
Chr6:31266207	HLA-C region	CA/TG	HLA-C_v8	rs386698994	MNV
Chr6:31274380	HLA-C region	T/C	HLA-Cw6_LD4	rs9264942	0.3654
Chr6:31274441	HLA-C region	G/A	HLA-C	rs9468942	0.0162
Chr6:31274449	HLA-C region	C/A	HLA-C_v9	rs35647108	0.0671
Chr6:31274513	HLA-C region	A/G	HLA-C_v10	rs6931873	0.2109
Chr6:31274518	HLA-C region	T/TCGGGGAG-TCCAG-CAGGTCC	HLA-C_v11	rs28383849	INS
Chr6:31274520	HLA-C region	C/CGGGAG-TCCAG-CAGGTCCCC	HLA-C	rs370331751	INS
Chr6:31274521	HLA-C region	G/A	HLA-C	rs9264943	0.1126
Chr6:31274555	HLA-C region	C/T	HLA-Cw6_LD5	rs10484554	0.1442
Chr6:31274580	HLA-C region	C/G	HLA-C_v12	rs184149624	0.0217
Chr6:31274582	HLA-C region	A/G	HLA-C_v13	rs9348865	0.3769
Chr6:31274584	HLA-C region	AA/A	HLA-C_v14	rs147538049	0.0439
Chr6:31274586	HLA-C region	A/G	HLA-C_v15	rs9348865	0.3769
Chr6:31274619	HLA-C region	A/G	HLA-C_v16	rs9264944	0.2350
Chr6:31274627	HLA-C region	G/A	HLA-C	rs9264945	0.0423
Chr6:31274630	HLA-C region	G/A	HLA-C	rs118169956	0.0156
Chr6:31274634	HLA-C region	T/C	HLA-C_v17	rs9264946	0.2053
Chr6:31274647	HLA-C region	G/A	HLA-C	rs9264947	0.1084
Chr6:31274661	HLA-C region	T/C	HLA-C	rs3132481	0.1025
Chr6:31274666	HLA-C region	C/T	HLA-C	rs76703505	0.0875
Chr6:31274677	HLA-C region	T/G	HLA-C	rs3132480	0.1020
Chr6:31274693	HLA-C region	G/A	HLA-C	rs3094691	0.4678
Chr6:31324742	HLA-B	T/C	HLA-B_v1	rs147324178	0.0000
Chr6:31324742	HLA-B	TG/CC	HLA-B_v2	rs796503852	MNV
Chr6:31324756	HLA-B	G/A	HLA-B_v3	rs41552714	0.0449
Chr6:31324788	HLA-B	T/C	HLA-B_v4	rs9266193	0.3105
Chr6:31324788	HLA-B	TCA/CCG	HLA-B_v5	rs796610180	MNV
Chr6:31324790	HLA-B	A/G	HLA-B_v6	rs9266194	0.0000
Chr6:31324819	HLA-B	C/T	HLA-B_v7	rs41557213	0.0149
Chr6:31324829	HLA-B	GG/CA	HLA-B_v8	rs9266196	0.0118
Chr6:31324938	HLA-B	C/T	HLA-B_v9	rs114811870	0.0502
Chr6:31324953	HLA-B	C/T	HLA-B_v10	rs9266207	0.0000
Chr6:31324953	HLA-B	CTC/TTT	HLA-B_v11	rs386699132	MNV
Chr6:31324996	HLA-B	G/C	HLA-B_v12	rs2596490	0.4462
Chr6:31325001	HLA-B	G/A	HLA-B_v13	rs151341073	0.0004
Chr6:31325004	HLA-B	G/A	HLA-B_v14	rs9266209	0.4120
Chr6:31325023	HLA-B	T/C	HLA-B_v15	rs9266210	0.2541
Chr6:31325023	HLA-B	TATCCAAT/CA CCCGAC	HLA-B_v16	rs796100503	MNV
Chr6:31325025	HLA-B	T/C	HLA-B_v17	rs9266211	0.4631
Chr6:31325028	HLA-B	A/G	HLA-B_v18	rs2596489	0.1857
Chr6:31325028	HLA-B	AAT/GAC	HLA-B_v19	rs796742686	MNV
Chr6:31325030	HLA-B	T/C	HLA-B_v20	rs2596488	0.1554
Chr6:31325049	HLA-B	G/A	HLA-B_v21	rs9266214	0.0209
Chr6:31325056	HLA-B	C/T	HLA-B_v22	rs2596487	0.1829

Chr6:31344471	FGFR3P1 region	C/G	FGFR3P1 region_v4	rs3997983	0.3476
Chr6:31344484	FGFR3P1 region	C/T	FGFR3P1 region_v5	rs114654060	0.0217
Chr6:31344490	FGFR3P1 region	G/A	FGFR3P1 region_v6	rs3957111	0.1235
Chr6:31344511	FGFR3P1 region	G/T	FGFR3P1 region_v1	rs28366075	0.0897
Chr6:31344571	FGFR3P1 region	T/G	FGFR3P1 region_v2	rs28366076	0.0877
Chr6:31344583	FGFR3P1 region	A/G	FGFR3P1 region_v3	rs13202464	0.0696
Chr6:31344614	FGFR3P1 region	C/T	FGFR3P1 region_v7	rs138591337	0.0055
Chr6:31344626	FGFR3P1 region	G/C	FGFR3P1 region_v8	rs9266596	0.2264
Chr6:31344657	FGFR3P1 region	C/A	FGFR3P1 region_v9	rs2844546	0.2931
Chr6:31344670	FGFR3P1 region	C/T	FGFR3P1 region_v10	rs2523637	0.3456
Chr6:31344778	FGFR3P1 region	C/G	FGFR3P1 region_v11	rs143610460	0.0001
Chr6:31361897	MICA region	A/C	MICA region_v2	rs2523473	0.3505
Chr6:31361974	MICA region	T/C	MICA region_v3	rs34821683	0.2279
Chr6:31361987	MICA region	C/T	MICA region_v4	rs35026345	0.3019
Chr6:31362010	MICA region	G/A	MICA region_v5	rs34464243	0.2723
Chr6:31362069	MICA region	T/G	MICA region_v6	rs67284927	0.1341
Chr6:31362120	MICA region	G/A	MICAregion_v1	rs66609536	0.2539
Chr6:31362159	MICA	C/T	MICA_v4	rs2428476	0.0879
Chr6:31362166	MICA	C/T	MICA_v5	rs28366116	0.0879
Chr6:31362179	MICA	G/A	MICA_v6	rs1052414	0.1787
Chr6:31362181	MICA	T/C	MICA_v7	rs1052413	0.1382
Chr6:31362207	MICA	T/C	MICA_v8	rs1052409	0.2169
Chr6:31362214	MICA	G/A	MICA_v9	rs148280868	0.0219
Chr6:31376928	MICA	T/C	**MICA_v1**	rs2523497	0.4312
Chr6:31376989	MICA	A/G	MICA_v10	rs12660741	0.0275
Chr6:31377047	MICA	C/T	MICA_v2	rs6910087	0.1305
Chr6:31377086	MICA	C/G	MICA_v3	rs528265306	0.0070
Chr6:31377100	MICA	A/G	MICA_11	rs3032981	0.1684
Chr6:31431636	HCP5	T/A	HCP5_v4	rs143344109	0.0013
Chr6:31431666	HCP5	G/T	HCP5_v5	rs749856170	0.0001
Chr6:31431691	HCP5	G/T	HCP5_v6	rs2255221	0.0818
Chr6:31431723	HCP5	G/A	HCP5_v7	rs2255223	0.0415
Chr6:31431757	HCP5	A/G	HCP5_v8	rs11752262	0.0714
Chr6:31431780	HCP5	T/G	HCP5_v1	rs2395029	0.0359
Chr6:31431813	HCP5	A/G	HCP5_v9	rs3130907	0.1051
Chr6:31431820	HCP5	C/T	HCP5_v2	rs2243621	0.1611
Chr6:31431874	HCP5	G/T	HCP5_v3	rs2395030	0.0474
Chr6:31431911	HCP5	A/G	HCP5_v10	rs78490730	0.0017
Chr6:31461372	MICB-DT	A/T	MICB-DT_v1	rs2507971	0.6052
Chr6:31461442	MICB-DT	T/C	MICB-DT_v3	rs372175088	0.0005
Chr6:31461492	MICB-DT	G/C	MICB-DT_v2	rs9267325	0.1409
Chr6:31461509	MICB-DT	G/T	MICB-DT_v4	rs2516413	0.2891
Chr6:31461558	MICB-DT	T/C	MICB-DT_v5	rs3094006	0.2653
Chr6:31461608	MICB-DT	C/T	MICB-DT_v6	rs2534687	0.3360
Chr6:31461613	MICB-DT	T/C	MICB-DT_v7	rs2252937	0.0698

Skin barrier function					
**Genome position**	**Gene**	**REF/ALT**	**SNP name**	**dbSNP ID**	**MAF**
Chr1:152549876	LCE region	C/T	LCE_v	rs16834215	0.0096
Chr1:152550018	LCE region	T/G	LCE_v1	rs4085613	0.6370
Chr1:152550103	LCE region	T/A,C	LCE_v	rs56038709	0.0000
Chr1:152551276	LCE3B region	A/G	LCE3B	rs4112788	0.6369
chr1:152551325	LCE3B region	C/T	LCE3B_v2	rs4112787	0.3505
chr1:152551547	LCE3D region	A/G	LCE3D	rs61813877	0.3435
Chr1:152590187	LCE region	C/T	LCE_v2	rs6677595	0.6361
Chr1:152590352	LCE3A-B intergenic region	G/A	LCE3A-B_v3	rs149178120	0.0036
Chr1:152590444	LCE3A-B intergenic region	G/A	LCE3A-B_v4	rs6701307	0.3916
Chr1:152590886	LCE3A-B intergenic region	T/C	LCE3A-B_v5	rs4845447	0.3921
Chr1:152590890	LCE3A-B intergenic region	G/A	**LCE3A-B_v2**	rs6701730	0.2454
Chr1:152590933	LCE3A-B intergenic region	G/A	LCE3A-B_v6	rs772160083	0.0003
Chr1:152590943	LCE3A-B intergenic region	T/C	LCE3A-B_v7	rs4845448	0.3244
Chr1:152590955	LCE3A-B intergenic region	T/C	LCE3A-B_v8	rs73019274	0.0068
Chr1:152591024	LCE3A-B intergenic region	T/A	LCE3A-B_v9	rs4845449	0.3925
Chr1:152591142	LCE region	A/C	LCE_v3	rs1886734	0.6369
Chr1:152591200	LCE3A-B intergenic region	T/C	LCE3A-B_v10	rs4845450	0.3881
Chr1:152591953	LCE3A-B intergenic region	G/C	LCE3A-B_v11	rs4845453	0.4916
Chr1:152591964	LCE3A-B intergenic region	A/G	LCE3A-B_v12	rs112857972	0.0136
Chr1:152592184	LCE region	C/T	LCE_v4	rs4845454	0.6376
Chr1:152593292	LCE3A-B intergenic region	G/A	**LCE3A-B_v1**	rs12030223	0.2539
Chr1:152593307	LCE3A-B intergenic region	G/A	LCE3A-B_v13	rs6662989	0.3599
Chr1:152593437	LCE3A-B intergenic region	T/C	LCE3A-B_v14	rs11205044	0.3931
Chr1:152593440	LCE3A-B intergenic region	A/G	LCE3A-B_v15	rs59980392	0.0053
Chr1:152593444	LCE3A-B intergenic region	T/C	LCE3A-B_v16	rs12027820	0.2643
Chr1:152593549	LCE region	C/T	LCE_v5	rs10888503	0.6442
Chr1:152593551	LCE3A-B intergenic region	T/C	LCE3A-B_v17	rs186644005	0.0112
Chr1:152778443	LCE1C	G/T	LCE1C_v2	rs12025125	0.1371
Chr1:152778526	LCE1C	C/T	LCE1C	rs6701216	0.1467
Chr1:152778558	LCE1C	C/T	LCE1C_v3	rs6701221	0.1515
Chr1:152778576	LCE1C	G/A	LCE1C_v4	rs4845488	0.4681
Chr1:152778584	LCE1C	C/T	LCE1C_v5	rs192573057	0.0095
Chr6:31084034	CDSN	C/T	CDSN_v8	rs117951780	0.0107
Chr6:31084048	CDSN	A/G	CDSN_v9	rs3094216	0.2195
Chr6:31084075	CDSN	G/C	CDSN_v10	rs3130982	0.4899
Chr6:31084147	CDSN	G/T	CDSN_v11	rs369037215	0.0001
Chr6:31084163	CDSN	A/G	CDSN_v1	rs3132554	0.5235
Chr6:31084170	CDSN	A/C	**CDSN_v2**	rs1042127	0.1935
Chr6:31084191	CDSN	T/C	CDSN_v3	rs33941312	0.0249
Chr6:31084288	CDSN	T/C	CDSN_v4	rs1042126	0.5185
Chr6:31084333	CDSN	G/A	CDSN_v12	rs36097319	0.0017
Chr6:31084627	CDSN	C/T	CDSN_v13	rs150163828	0.0004

Chr6:31084639	CDSN	C/T	**CDSN_v5**	rs4713436	0.1821
Chr6:31084684	CDSN	G/A	CDSN_v14	rs3094215	0.4700
Chr6:31084714	CDSN	G/A	CDSN_v15	rs145637055	0.0012
Chr6:31084723	CDSN	G/A	CDSN_v16	rs117764398	0.0004
Chr6:31084787	CDSN	A/G	CDSN_v6	rs707913	0.2139
Chr6:31084792	CDSN	C/T	CDSN_v7	rs3130983	0.5186
Chr6:31110119	CCHCR1 region	A/G	CCHCR1_v8	rs183837992	0.0019
Chr6:31110229	CCHCR1	G/A	CCHCR1_v9	rs116109529	0.0058
Chr6:31110391	CCHCR1	G/C	CCHCR1_v1	rs1576	0.3461
Chr6:31112737	CCHCR1	C/A	CCHCR1_v2	rs130079	0.2491
Chr6:31112823	CCHCR1	T/C	CCHCR1_v10	rs2073720	0.0094
Chr6:31112899	CCHCR1	C/T	CCHCR1_v11	rs3094226	0.2305
Chr6:31112925	CCHCR1	C/T	**CCHCR1_v3**	rs2073719	0.2393
Chr6:31114182	CCHCR1	A/G	CCHCR1_v4	rs746647	0.3220
Chr6:31114335	CCHCR1	A/G	CCHCR1_v12	rs2240066	0.0434
Chr6:31114358	CCHCR1	T/G	CCHCR1_v13	rs369540929	0.0019
Chr6:31114448	CCHCR1	CC/TT	CCHCR1_v5	rs375143475	none
Chr6:31114449	CCHCR1	C/T	CCHCR1_v14	rs2240065	0.1921
Chr6:31122315	CCHCR1	G/C	CCHCR1_v15	rs130066	0.4133
Chr6:31122330	CCHCR1	G/A	CCHCR1_v16	rs130077	0.1519
Chr6:31122393	CCHCR1	G/A	CCHCR1_v17	rs753814781	0.0000
Chr6:31122472	CCHCR1	C/T	CCHCR1_v18	rs576214578	0.0001
Chr6:31122482	CCHCR1	G/A	CCHCR1_v6	rs130076	0.2185
Chr6:31122500	CCHCR1	G/A	CCHCR1_v7	rs130065	0.2083
Chr6:31122502	CCHCR1	C/T	CCHCR1_v19	rs130075	0.0527
Chr6:31122521	CCHCR1	G/A	CCHCR1_v20	rs530074163	0.0000
Chr6:31122551	CCHCR1	C/T	CCHCR1_v21	rs146335956	0.0008
Chr6:31122564	CCHCR1	C/G	CCHCR1_v22	rs144885162	0.0179
Chr9:110816795	KLF4 region	T/C	KLF4_v2	rs1369190	0.3718
Chr9:110817020	KLF4 region	A/G	KLF4	rs10979182	0.4154

Innate immunity

Genome position	Gene	REF/ALT	SNP name	dbSNP ID	MAF
Chr2:113736206	IL36G	C/T	IL36G_v4	rs756860964	0.0000
Chr2:113736280	IL36G	A/G	IL36G_v5	rs758024889	none
Chr2:113736296	IL36G	G/T	IL36G _v1	rs28947206	0.0002
Chr2:113736325	IL36G	C/T	IL36G _v2	rs28947207	0.0002
Chr2:113739437	IL36G	T/C	IL36G_v6	rs187067341	0.0007
Chr2:113739532	IL36G	G/A	IL36G_v7	rs6743744	0.0639
Chr2:113739563	IL36G	T/C	IL36G _v3	rs28947211	0.0003
Chr2:113739597	IL36G	T/A	IL36G_v8	rs139031255	0.0012
Chr2:163123826	IFIH1	C/A	IFIH1_v2	rs74162090	0.0001
Chr2:163123842	IFIH1	G/A	IFIH1_v3	rs74162089	0.0019
Chr2:163124051	IFIH1	C/T	IFIH1	rs1990760	0.6002
Chr6:31540071	LTA	G/A	LTA_v1	rs1800683	0.3257
Chr6:31540141	LTA	AG/CG	LTA_v4	rs2239704	0.4097
Chr6:31540313	LTA	A/G	LTA_v2	rs909253	0.3326
Chr6:31542308	LTA region	T/C	LTA_v5	rs1799964	0.2115
Chr6:31542476	LTA region	C/A	LTA_v6	rs1800630	0.1526
Chr6:31542482	LTA region	C/T	LTA_v3	rs1799724	0.1149
Chr6:31542533	LTA region	C/T	LTA_v7	rs4248158	0.0153
Chr8:7272329	DEFB4B	C/T	DEFB4B_v3	rs2737531	0.3956
Chr8:7272356	DEFB4B	C/T	DEFB4B_v4	rs2737913	0.0593
Chr8:7272439	DEFB4B	G/A	DEFB4B_v1	rs2740091	0.1781
Chr8:7273050	DEFB4B	T/C	DEFB4B_v2	rs73661358	0.1517
Chr9:32534685	DDX58 region	A/G	DDX58_v3	rs73644984	0.0083
Chr9:32534714	DDX58 region	T/G	DDX58_v1	rs34085293	0.1410
Chr9:32534851	DDX58 region	G/A	DDX58_v2	rs657454	0.6309
Chr12:56737973	STAT2	A/G	STAT2	rs2066808	0.0671
Chr19:10469975	TYK2	A/C	TYK2_v1	rs12720356	0.0883
Chr19:10470085	TYK2	G/A	TYK2_v5	rs777784293	0.0002
Chr19:10472933	TYK2	A/G	TYK2_v2	rs280519	0.5167
Chr19:10473033	TYK2	C/T	TYK2_v6	rs758682935	0.0001
Chr19:10473050	TYK2	C/T	TYK2_v7	rs142576987	0.0002
Chr19:10473076	TYK2	G/A	TYK2_v8	rs774211784	0.0000
Chr19:10475649	TYK2	C/T	TYK2_v3	rs2304255	0.0758
Chr19:10475652	TYK2	C/A	TYK2_v4	rs2304256	0.2850
Chr19:10475760	TYK2	G/A	TYK2_v9	rs12720270	0.1634
Chr20:30045269	DEFB123 region	G/A	DEFB123_v2	rs559204339	0.0003
Chr20:30045393	DEFB123 region	G/A	DEFB123	rs6088273	0.6282

Cytokine-dependent pathways and T-cell signaling

Genome position	Gene	REF/ALT	SNP name	dbSNP ID	MAF
Chr1:12252892	TNFRSF1B	C/T	TNFRSF1B_v4	rs1768642	0.2244
Chr1:12252955	TNFRSF1B	T/G	TNFRSF1B_v1	rs1061622	0.2367
Chr1:12253062	TNFRSF1B	G/A	TNFRSF1B_v5	rs5746026	0.0314
Chr1:12267099	TNFRSF1B	C/T	TNFRSF1B_v6	rs150675043	0.0005
Chr1:12267102	TNFRSF1B	A/G	TNFRSF1B_v7	rs371676482	0.0005
Chr1:12267265	TNFRSF1B	A/G	TNFRSF1B_v2	rs1061624	0.5554
Chr1:12267270	TNFRSF1B	T/G	TNFRSF1B_v8	rs5030792	0.0410
Chr1:12267292	TNFRSF1B	C/T	TNFRSF1B_v3	rs3397	0.6393
Chr1:25292799	RUNX3 region	C/T	RUNX3_v2	rs554614108	0.0016
Chr1:25292896	RUNX3 region	A/G	RUNX3_v3	rs767088722	0.0001
Chr1:25293084	RUNX3 region	T/C	RUNX3	rs7536201	0.5437
Chr1:67600686	IL23R region	G/T	IL23R_v1	rs12044149	0.2526
Chr1:67611613	IL23R	G/A	IL23R_v2	rs4655683	0.3397
Chr1:67658723	IL23R	T/C	IL23R_v8	rs12567033	0.0001
Chr1:67658769	IL23R	C/T	IL23R_v9	rs12562213	0.0273
Chr1:67658803	IL23R	G/A	IL23R_v3	rs72676067	0.2915
Chr1:67658954	IL23R	T/C	IL23R_v10	rs72676069	0.3016
Chr1:67670133	IL23R	T/G	IL23R_v11	rs1004820	0.3989
Chr1:67670213	IL23R	G/A	IL23R_v4	rs1004819	0.2987
Chr1:67694010	IL23R	G/A	IL23R_v12	rs374068944	0.0001
Chr1:67694202	IL23R	A/G	IL23R_v5	rs2201841	0.3059
Chr1:67705900	IL23R	G/A	IL23R_v6	rs41313262	0.0160
Chr1:67705958	IL23R	G/A	IL23R_v7	rs11209026	0.0656
Chr2:204738725	CTLA4	C/T	CTLA4_v1	rs231721	0.0083
Chr2:204738919	CTLA4	G/A	CTLA4_v2	rs3087243	0.4366
Chr2:204738938	CTLA4 region	G/A	CTLA4 region	rs11571319	0.1652
Chr3:101575779	NFKBIZ	G/A	NFKBIZ	rs537411494	0.0020
Chr3:101575882	NFKBIZ	T/C	NFKBIZ	rs595788	0.2838
Chr3:101575902	NFKBIZ	C/T	NFKBIZ	rs7628891	0.0281
Chr3:101576029	NFKBIZ	T/TACTTTTA-GAAA-GCTTTAA-TAACC	NFKBIZ_v1	rs3217713	0.7714
Chr3:101615580	NFKBIZ region	C/G	NFKBIZ_v4	rs534286886	0.0001
Chr3:101615625	NFKBIZ region	T/C	NFKBIZ_v5	rs9818678	0.4951
Chr3:101615801	NFKBIZ region	A/G	NFKBIZ_v6	rs114576100	0.0107
Chr3:101615826	NFKBIZ region	G/T	NFKBIZ_v2	rs4683946	0.1998
Chr3:101663317	NFKBIZ region	A/G	NFKBIZ_v7	rs9881690	0.3677
Chr3:101663323	NFKBIZ region	A/G	NFKBIZ_v8	rs77371624	0.0215
Chr3:101663386	NFKBIZ region	C/T	NFKBIZ_v9	rs7625614	0.4198
Chr3:101663555	NFKBIZ region	A/G	NFKBIZ_v3	rs7637230	0.2087
Chr3:124811314	SLC12A8	G/A	SLC12A8	rs651630	0.4786
Chr5:150466997	TNIP1	C/T	TNIP1_v3	rs557528520	0.0019
Chr5:150467130	TNIP1	T/G	TNIP1_v4	rs140378407	0.0049
Chr5:150467170	TNIP1	T/C	TNIP1_v5	rs2233279	0.4209
Chr5:150467189	TNIP1	G/C	TNIP1_v1	rs2233278	0.0507
Chr5:150476004	TNIP1 region	T/C	TNIP1_v2	rs1024995	0.1124
Chr5:150476129	TNIP1 region	C/T	TNIP1_v6	rs11738559	0.1709
Chr5:150476200	TNIP1 region	T/A	TNIP1_v7	rs770810925	0.0000
Chr5:158742785	IL12B	A/G	IL12B_v4	rs542242777	0.0002
Chr5:158742950	IL12B	T/G	IL12B_v1	rs3212227	0.2029
Chr5:158750544	IL12B	G/A	IL12B_v5	rs140257668	0.0053
Chr5:158750761	IL12B	G/A	IL12B_v6	rs183593461	0.0016

Chr5:158750769	IL12B	C/T	IL12B_v2	rs3213094	0.2048
Chr5:158759900	IL12B region	A/G	IL12B_v3	rs2546890	0.4861
Chr5:158759915	IL12B region	G/A	IL12B_v7	rs559446512	0.0000
Chr5:159912418	MIR146A	C/G	MIR146A	rs2910164	0.7600
Chr6:31542963	TNF-α region	G/A	TNFA	rs1800750	0.0157
Chr6:31543031	TNF-α region	G/A	TNFA_v1	rs1800629	0.1624
Chr6:31543101	TNF-α region	G/A	TNFA_v2	rs361525	0.0529
Chr6:31543581	TNF-α	AG/A	TNFA	rs766034823	0.0000
Chr6:31543758	TNF-α	G/A	TNFA	rs3093661	0.0319
Chr6:31543825	TNF-α	A/T	TNFA	rs4645839	0.0031
Chr6:31543827	TNF–α	G/A	**TNFA_v3**	rs1800610	0.0869
Chr6:31544189	TNF-α	A/G	TNFA	rs3093662	0.0779
Chr6:31543943	TNF–α	G/GTGAA	TNFA_v4	rs374501689	DEL
Chr6:32814820	TAP1	G/T	TAP1_v1	rs55865470	0.0295
Chr6:32814902	TAP1	C/T	TAP1_v2	rs41551515	0.0294
Chr6:32814942	TAP1	C/T	TAP1_v3	rs1057149	0.0335
Chr6:32814975	TAP1	T/C	TAP1_v4	rs1135216	0.1500
Chr6:32815025	TAP1	G/A	TAP1_v5	rs111511638	0.0487
Chr6:52101739	IL17F	T/C	IL17F_v1	rs56499381	0.0473
Chr6:52101758	IL17F	C/T	IL17F_v2	rs11465553	0.0407
Chr6:52101768	IL17F	G/A	IL17F_v4	rs148267766	0.0001
Chr6:52101844	IL17F	T/C	IL17F_v3	rs2397084	0.0942
Chr6:111577673	TRAF3IP2 region	A/G	TRAF3IP2_v5	rs116561834	0.0031
Chr6:111577761	TRAF3IP2 region	A/G	TRAF3IP2_v1	rs71562288	0.0917
Chr6:111673714	TRAF3IP2 region	T/C	TRAF3IP2_v2	rs240993	0.7282
Chr6:111673756	REV3L	C/T	REV3L	rs779554762	0.0004
Chr6:111913070	TRAF3IP2	G/A	TRAF3IP2_v6	rs13190932	0.0579
Chr6:111913262	TRAF3IP2 region	C/T	TRAF3IP2_v3	rs33980500	0.0786
Chr6:111922503	TRAF3IP2	G/C	TRAF3IP2_v7	rs76228616	0.0406
Chr6:111922720	TRAF3IP2 region	A/G	TRAF3IP2_v4	rs13210247	0.0662
Chr6:138195991	TNFAIP3	A/G	TNFAIP3_v4	rs146534657	0.0014
Chr6:138196066	TNFAIP3	T/G	TNFAIP3_v1	rs2230926	0.0348
Chr6:138196151	TNFAIP3	G/A	TNFAIP3_v5	rs140424499	0.0006
Chr6:138197824	TNFAIP3	C/T	TNFAIP3_v2	rs582757	0.7371
Chr6:138199377	TNFAIP3	A/C	TNFAIP3_v6	rs564537694	0.0001
Chr6:138199417	TNFAIP3	G/T	TNFAIP3_v3	rs610604	0.6745
Chr9:117552885	TNFSF15	T/C	TNFSF15_v1	rs3810936	0.6912
Chr9:117552954	TNFSF15	A/C	TNFSF15_v5	rs187538454	0.0000
Chr9:117558703	TNFSF15	C/T	TNFSF15_v2	rs6478108	0.6693
Chr9:117566440	TNFSF15	A/G	TNFSF15_v3	rs4263839	0.6850
Chr9:117568766	TNFSF15 region	A/G	TNFSF15_v4	rs6478109	0.6794
Chr12:6450845	TNFRSF1A	C/T	TNFRSF1A_v2	rs151344626	0.0011
Chr12:6450871	TNFRSF1A	C/T	TNFRSF1A_v3	rs75304026	0.0001
Chr12:6450945	TNFRSF1A	T/C	TNFRSF1A	rs767455	0.4326
Chr16:11365500	SOCS1 region	C/T	SOCS1	rs367569	0.2777
Chr16:11365416	SOCS1 region	G/A	SOCS1_v2	rs431918	0.1712
Chr16:30942521	FBXL19	T/A	FBXL19_v1	rs79714718	0.0032
Chr16:30942595	FBXL19	C/T	FBXL19_v2	rs145623212	0.0007
Chr16:30942625	FBXL19	G/A	FBXL19_v3	rs10782001	0.3643
Chr17:26106675	NOS2	A/G	NOS2_v1	rs4795067	0.3519
Chr17:26124699	NOS2	T/C	NOS2_v3	rs16949	0.2221
Chr17:26124908	NOS2	G/A	NOS2_v2	rs28998802	0.1520
Chr17:78157811	CARD14	T/G	CARD14	rs146214639	0.0023
Chr21:36470865	RUNX1 region	C/T	RUNX1	rs8128234	0.2114

Chr21:36470574	RUNX1 region	T/C	RUNX1_v2	rs2834760	0.2097
Chr22:17565035	IL17RA region	G/A	IL17RA	rs4819554	0.8084
Chr22:17564736	IL17RA region	A/G	IL17RA_v2	rs41524752	0.0088
Chr22:17564907	IL17RA region	G/A	IL17RA_v3	rs4819553	0.1882
Chr22:17565013	IL17RA region	A/G	IL17RA_v4	rs4819958	0.1957

The SNP panel was composed of n = 417 SNPs located in psoriasis-risk genes. SNP-carrying genes were classified accordingly to their functions (antigen presentation, skin barrier, innate immune responses and cytokine-dependent signalling).
Notes*:* Genome position on Chromosome (Chr), UCSC Genome Browser hg19 assembly; Gene name; REF/ALT, reference base / alteration base; SNP, single-nucleotide polymorphism; dbSNP ID, data base SNP identification number at NCBI; MAF, minor allele frequency from dbGaP in European population; rs, reference SNP ID number; ERAP1, endoplasmic reticulum aminopeptidase 1; PSORS1C3, Psoriasis Susceptibility 1 Candidate 3; HLA, Human Leukocyte Antigen; FGFR3P1, fibroblast growth factor receptor 3 pseudogene 1; MICA, MHC class I polypeptide-related sequence A; HCP5, HLA complex P5; MICB-DT, MHC class I polypeptide-related sequence B- Divergent Transcription; LCE, late cornified envelope; CDSN, corneodesmosin; CCHCR1, coiled-coil alpha-helical rod protein 1; KLF4, Krüppel-Like Factor 4; IL, interleukin; IFIH1, Interferon Induced With Helicase C Domain 1; LTA, Lymphotoxin Alpha; DEFB, Defensin Beta; DDX58, DExD/H-Box Helicase 58; STAT2, signal transducer and activator of transcription 2; TYK2, tyrosine kinase 2; TNFRSF, Tumor necrosis factor receptor superfamily; RUNX, Runt-related transcription factor; Cytotoxic T-Lymphocyte Antigen 4, CTLA 4; NFKBIZ, NF-kB inhibitor zeta; Solute Carrier Family 12 Member 8, SLC12A8; TNIP1, TNFAIP3 Interacting Protein 1; MIR146A, MicroRNA 146a; protein reversionless 3-like, REV3L; TRAF3IP2, TRAF3 interacting protein 2; TNFAIP3, TNF alpha induced protein 3; TNFSF15, TNF superfamily member 15; SOCS1, suppressor of cytokine signaling 1; NOS2, Nitric Oxide Synthase 2; CARD14, Caspase Recruitment Domain Family Member 14; LD, linkage disequilibrium; MNV, Multi-nucleotide variants; INS, insertion. SNPs significantly associating with response to ustekinumab are indicated in font bold.

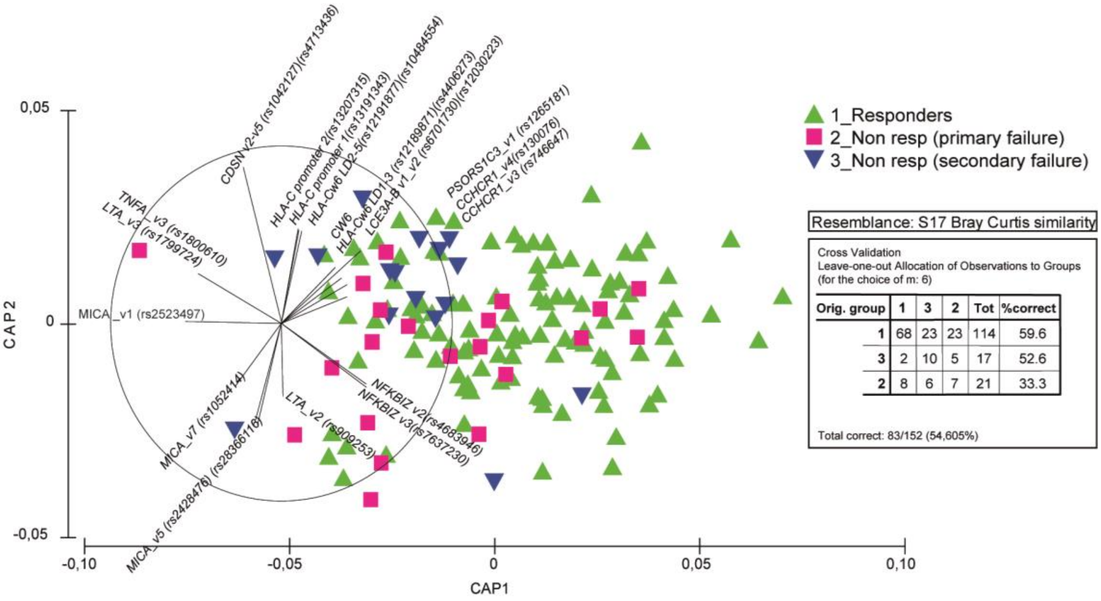

Orig. group	1	3	2	Tot	%correct
1	68	23	23	114	59.6
3	2	10	5	17	52.6
2	8	6	7	21	33.3

Figure S1. SNPs patterns in patient populations clustered based on their clinical response rate to ustekinumab. Analysis of SNP patterns by CAP performed on patient populations clustered based on their clinical response rate to ustekinumab: responders up to 2-year treatment (green triangles) (group 1, n=114), non-responders or only partially responders for primary failure (fuchsia squares) (group 2, n=21) or responders losing responsiveness over time for secondary failure (blue downward triangles) (group 3, n=17). The length of each vector line corresponds to the strength of the correlation and direction for each. Distinctness of the three patient groups was assessed using leave-one-out allocation success. 83 out of 152 (54,605%) patients were correctly allocated. Only the most relevant correlations were considered as valuable and included in the plot.

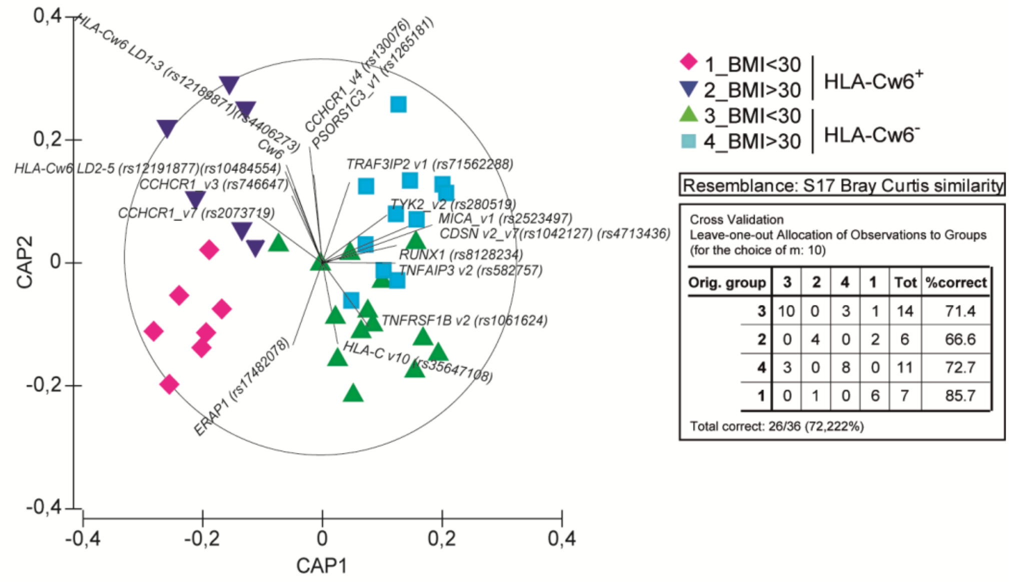

Orig. group	3	2	4	1	Tot	%correct
3	10	0	3	1	14	71.4
2	0	4	0	2	6	66.6
4	3	0	8	0	11	72.7
1	0	1	0	6	7	85.7

Figure S2. SNPs patterns in patient populations clustered based on BMI values and HLA-Cw6 allele presence/absence. SNP patterns were identified by CAP performed on non-responder patient populations (n=36 clustered based on BMI values and presence or absence of HLA-Cw6 allele. CAP ordination plot shows a significant clustering of psoriatic patients belonging to HLA-Cw6^{+} and HLA-Cw6^{-} groups along *x*-axis and to BMI <30 or >30 groups along *y*-axis. Four established subgroups are showed in the plot: 1_HLA-Cw6^{+} BMI <30 (fuchsia diamonds), 2_HLA-Cw6^{+} BMI >30 (blue downward triangles), 3_HLA-Cw6^{-} BMI <30 (green triangles) and 4_HLA-Cw6^{-} BMI <30 (light blue squares). 26 out of 36 (72,222%) patients were correctly allocated. Only the most relevant correlations were considered as valuable and included in the plot.
